# Theoretical investigation of the A^1^Π–X^1^Σ^+^, B^1^Σ^+^–X^1^Σ^+^, C^1^Σ^+^–X^1^Σ^+^, and E^1^Π–X^1^Σ^+^ transitions of the CO molecule†

**DOI:** 10.1039/d4cp03418j

**Published:** 2024-12-27

**Authors:** Malathe Khalil, Salman Mahmoud, Ryan P. Brady, Mubarak Almehairbi, Marko Gacesa, Sergei N. Yurchenko, Jonathan Tennyson, Amal Al Ghaferi, Nayla El-Kork

**Affiliations:** a Department of Mechanical Engineering, Khalifa University Abu-Dhabi United Arab Emirates; b Physics Department, Khalifa University Abu-Dhabi United Arab Emirates; c Department of Physics and Astronomy, University College London London WC1E 6BT UK j.tennyson@ucl.ac.uk; d Chemistry Department, Khalifa University Abu-Dhabi United Arab Emirates; e Planetary Science Center, Khalifa University Abu-Dhabi United Arab Emirates nayla.elkork@ku.ac.ae; f Rabdan Academy Abu Dhabi United Arab Emirates aalghafri@ra.ac.ae

## Abstract

The spectrum of carbon monoxide is important for astrophysical media, such as planetary atmospheres, interstellar space, exoplanetary and stellar atmospheres; it also important in plasma physics, laser physics and combustion. Interpreting its spectral signature requires a deep and thorough understanding of its absorption and emission properties. A new accurate spectroscopic model for the ground and electronically-excited states of the CO molecule computed at the aug-cc-pV5Z *ab initio* CASSCF/MRCI+Q level is reported. Detailed investigation of the A^1^Π–X^1^Σ^+^, B^1^Σ^+^–X^1^Σ^+^, C^1^Σ^+^–X^1^Σ^+^, and E^1^Π–X^1^Σ^+^ band systems is presented consisting of calculated potential energy curves as well as permanent and transition dipole moment curves. The B^1^Σ^+^ and C^1^Σ^+^ states are characterized by having multiple avoided crossings which are diabatized to obtain an accurate electronic structure model. The results are validated by comparing our computed spectra with various high-resolution spectroscopy experiments. To the best of our knowledge, this is the first systematic theoretical spectroscopic study of highly excited states of the CO molecule.

## Abstract 2

Potential energy and dipole moment curves for CO are calculated using a high level theory and diabatised. The resulting curves are used to compute electronic spectra of CO found in agreement with laboratory and astronomical observations.

## Graphical abstract

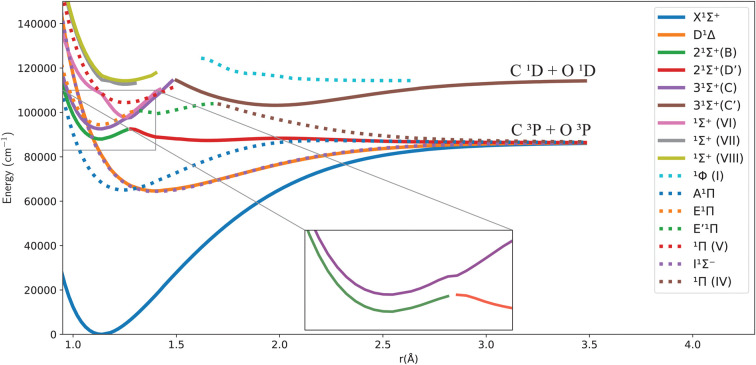

## 1 Introduction

After hydrogen, carbon monoxide is the second most abundant molecule in the Universe.^1,2^ It has been detected in Earth's atmosphere,^3^ comets,^4,5^ planetary atmospheres of Venus and Mars,^6,7^ interstellar clouds,^8^ circumstellar envelopes,^9^ and exoplanets,^10^ including HD 189733b^11^ and HD 209458b.^12^ CO is a common component of cool stars including the Sun.^13^ Studying the spectral signatures of the CO molecule can contribute to a deeper understanding of the chemical and structural composition of planetary and exoplanetary atmospheres as well as their evolution and approximate age.^10,14^ Electronic spectra of CO also have important signatures which are well-studied in plasma physics;^15–17^ indeed the names for several of the bands are named after their signature in plasmas. CO is also an important intermediary in combustion.

For these applications, developing an accurate theoretical spectroscopic model for CO is essential and allows analyses of specific emission and absorption bands involving its excited electronic states.^18^ For example, the fourth positive band system (A^1^Π–X^1^Σ^+^) and the Hopfield-Birge system (B^1^Σ^+^–X^1^Σ^+^ and C^1^Σ^+^–X^1^Σ^+^) are the main components of carbon monoxide's ultraviolet (UV) absorption spectrum.^4^ Moreover, the A^1^Π–X^1^Σ^+^ transition is commonly observed in plasmas^16^ and has been observed in Mars’ atmospheric spectrum^19^ and the red rectangle Nebula,^20^ while the B^1^Σ^+^–X^1^Σ^+^ and C^1^Σ^+^–X^1^Σ^+^ transitions have been observed in cometary comae, including Comet C/2001 A2 (LINEAR).^5^ The Rydberg series lying energetically above the CO dissociation limit are crucial for photodissociation studies, particularly the B^1^Σ^+^ and C^1^Σ^+^ states.^21^ The D′^1^Σ^+^ state also plays a vital role in the photodissociation of CO, with possible direct consequences on the escape of carbon from the Martian atmosphere, where photodissociation has been identified as the key exothermal production channel of atomic carbon.^22–24^

Developing accurate theoretical models to generate synthetic spectra and photodissociation requires precise calculations of excited electronic and rovibrational states. The electronic and rovibronic energy levels and line lists for CO are crucial in forward modeling for the characterization of the atmospheric emissions in the FUV region and enable the development of theoretical investigations of the planetary atmospheres.^25^ At conditions found in solar and stellar atmospheres, producing high-resolution spectra that account for highly excited vibrational and rotational (rovibrational) levels tailored to the temperature and pressure of the gas is vital. Available databases, such as the high-temperature molecular spectroscopic database (HITEMP),^26^ an extension of the high-resolution transmission molecular absorption database (HITRAN),^27^ and ExoMol^28^ include extensive and reliable line lists for rovibrational spectrum of CO which are regularly updated,^29^ but do not include ones for the excited electronic states of CO, in contrast, for example with NO for which good rovibronic line lists are available.^30,31^ Therefore, there is a pressing need to bridge these data and understanding gaps.

The theoretical investigation of CO electronic spectra goes back to 1981 when Cooper and Langhoff^32^ used self-consistent-field (SCF) and configuration-interaction (CI) methods to calculate the molecule's potential energy curves, with a large Slater basis set that was augmented with diffuse functions. In their calculations, the second and third ^1^Σ^+^ states were denoted the B^1^Σ^+^ and C^1^Σ^+^ states. They also predicted a fourth ^1^Σ^+^ state that crosses them. Subsequently, Cooper and Kirby^33–35^ calculated the adiabatic potential energy curves of CO using a Slater-type basis sets using a multi-configuration (MC)-SCF-CI method. They found that the B^1^Σ^+^ Rydberg state mentioned by Cooper and Langhoff^32^ actually belongs to a 2^1^Σ^+^ state, which has, in reality, a characteristic double-minimum potential. The inner well represents the B^1^Σ^+^ state, while the outer well corresponds to the D′^1^Σ^+^ valence state. The B^1^Σ^+^ state has been extensively investigated by Eidelsberg *et al.*^36^ in terms of band spectra. The D′ state had been studied both experimentally^37^ and theoretically.^38^ Similarly, they characterized the state 3^1^Σ^+^ with a double minimum, the inner well being represented by the Rydberg C^1^Σ^+^ state and the outer one by a C′^1^Σ^+^ state.^33^ Cooper and Kirby^35^ also found a slightly visible avoided crossing between the 2^1^Σ^+^ and the 3^1^Σ^+^ around 1.32 Å. Like Cooper and Kirby,^33,34^ Tchang-Brillet *et al.*^39^ later noted that the B^1^Σ^+^ state correlates adiabatically with D′^1^Σ^+^ giving rise to a double minimum adiabatic potential that they called the “BD′” potential. They stated that the double minimum detected by Cooper and Kirby results from “an avoided crossing between the diabatic potential curves of Rydberg and valence character”, and investigated the predissociation interaction between them, using a nonperturbative spectroscopic model based on a diabatic Rydberg–Klein–Rees (RKR) potential generated from the experimental spectroscopic constants. The diabatic representation also includes a crossing of the D′ state with both the B^1^Σ^+^ and C^1^Σ^+^ Rydberg states.

**Fig. 1:**
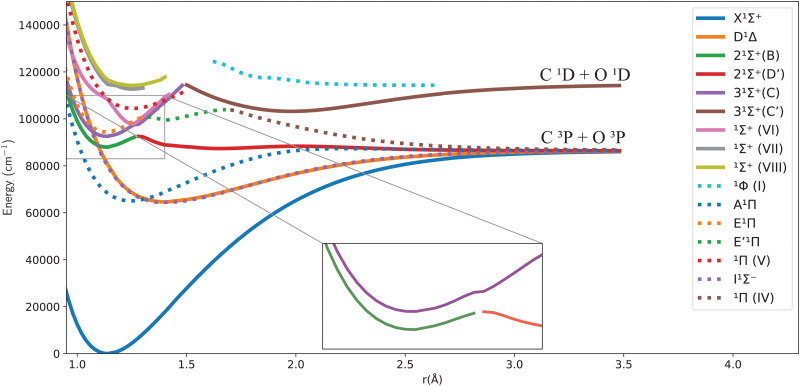
Potential energy curves for the singlet states of CO in the adiabatic representation.

**Fig. 2:**
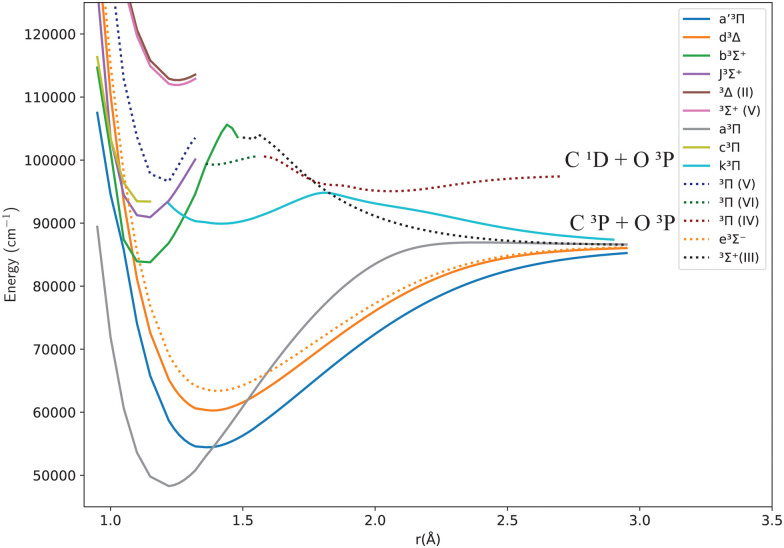
Potential energy curves for the triplet states of CO in the adiabatic representation.

Fig. 1 and 2 give an overview of the singlet and triplet states of CO considered here. These states undergo a complicated set of avoided crossings, as mentioned above, in particular, between the second, third, and fourth ^1^Σ^+^ states. We have denoted the first two as 2^1^Σ^+^ and 3^1^Σ^+^, as per Cooper and Kirby,^33,34^ while at the same time referring to the B^1^Σ^+^ and D′^1^Σ^+^ sections and C^1^Σ^+^ and C′^1^Σ^+^ sections with different colours, as per Tchang-Brillet *et al.*^39^ notation. For example, the legend reads 2^1^Σ^+^(B) for the portion of the 2^1^Σ^+^ state which refers to the Rydberg B^1^Σ^+^ state, while 2^1^Σ^+^(D′) refers to the valence D′^1^Σ^+^ region of the same state. Also, in the following sections of this work, a reference to the BD′ system implicitly means the 2^1^Σ^+^ state, while the CC′ system refers to the 3^1^Σ^+^ state.

One can notice from Fig. 1 that the potential barrier of the 2^1^Σ^+^ (or BD′) state is itself avoiding the upper 3^1^Σ^+^ (or CC′) state in the adiabatic representation. The 3^1^Σ^+^ state also undergoes an avoided crossing with the ^1^Σ^+^(VI) state, located at even higher energy. Given this complicated system, treating the avoided crossings correctly represents an important part of constructing a robust spectroscopic model. More discussion about them and their diabatization is presented below.

It is worth noting that Vázquez *et al.*^40^ used the SCF MRSD-CI method with the aug-cc-pVQZ basis set to calculate the potential energy curves of CO; they found an energy difference between the minima of the C^1^Σ^+^ and D′^1^Σ^+^ states of around 4748 cm^−1^; they also reported a literature value for the experimentally obtained energy difference of 2476 cm^−1^. Furthermore, they reported the potential barrier of D′ to be 1048 cm^−1^ above the dissociation limit C(^3^P) + O(^3^P) at 1.980 Å. They suggest that this feature occurs due to an avoided crossing with a (^1^Σ^+^) bound state that converges to the C(^1^D) + O(^1^D) asymptotic limit or to C(^3^P) + O(^3^P) without specifying the exact state. They also calculated *T*_e_ for the C′^1^Σ^+^ valence state of 104 127 cm^−1^ compared to the experimental *T*_e_ value of 102 207 cm^−1^.^41^

The first ^1^Σ^+^ valence state (D′^1^Σ^+^) causes strong predissociation and perturbations in the Rydberg states (^1^Σ^+^).^42^ The perturbations happen in the (*n*sσ) Rydberg states of the CO molecule and manifest as an energy shift in the lowest vibrational levels. In particular, the *ν* = 2 vibrational level of the B^1^Σ^+^ state is strongly affected by this perturbation due to electrostatic interaction with the D′^1^Σ^+^ valence state.^42^ The D′^1^Σ^+^ state has a weakly bound repulsive part that causes large changes in the vibrational and rotational parameters upon interaction with the B^1^Σ^+^ state, as well as significantly increased predissociation rates of the *ν* = 2 and *ν* = 3 vibrational energy levels. Strong coupling between the B^1^Σ^+^ and D′^1^Σ^+^ states leads to a ^1^Σ^+^ resonance in the absorption spectra. In general, the strength of the interaction varies as *n*^−3/2^, where *n* is the index of the state within the *n*sσ series.^41^ Some vibrational levels of the C′^1^Σ^+^ state are accessible from the *ν* = 0 vibrational level of the ground state at higher energies due to the interaction of the C′^1^Σ^+^ state with the B^1^Σ^+^ Rydberg state.^41^ The *ν* = 0 and *ν* = 1 levels of the B^1^Σ^+^ states have longer lifetimes than the higher vibrational levels and give rise to fluorescence to the A^1^Π state and the ground state.

The (2–0) B^1^Σ^+^–X^1^Σ^+^ band has a rotational bandwidth of around 1 to 2 cm^−1^ (FWHM),^43^ while the (3–0) band is diffuse, and the higher bands are even broader.^39,43^ Also, above 100 000 cm^−1^, the absorption of CO is complicated because the molecule undergoes photodissociation.^41^ Finally, the E^1^Π state is the second ^1^Π state and the first ^1^Π Rydberg state of the CO molecule. It has an experimentally determined minimum energy of 92 903 cm^−1^, which lies above that of the C^1^Σ^+^ state.^44^ It undergoes an avoided crossing with E′^1^Π at around 1.25 Å.^42^

In 2004 Eidelsberg *et al.*^41^ updated the diabatic potential energy curves published in 1992: the C′^1^Σ^+^ state minimum at 1.8 Å is obtained at 107 600 cm^−1^ above the ground state's minimum. Their *ab initio* calculations underestimate the well depth of the C′^1^Σ^+^ state by 5400 cm^−1^.^41^

Lu *et al.*^45^ calculated the low-lying states of CO in 2012, along with other singlet and triplet states converging to the first dissociation limit, using the cc-pV5Z and aug-cc-pV5Z basis sets and the CASSCF/MRCI+Q (complete active space self-consistent field/multi-reference configuration interaction + Davidson correction) method. They obtained an energy for the lowest vibrational level (*v* = 0) of the A^1^Π state of 756.5 cm^−1^, 3 cm^−1^ higher than the experimental value (753.5 cm^−1^). A synthetic spectrum for the A^1^Π–X^1^Σ^+^ band system was calculated by Cheng *et al.*,^46^ for which they used MRCI+Q with an aug-cc-pV5Z basis set to calculate the potential energy curves. The authors reported that they did not use a theoretical transition dipole moment (TDM), but instead used an indirect method based on experimental laser-induced fluorescence, to determine an empirical TDM function. Forty vibrational levels were calculated for the ground state (*ν*′′ = 0–39), and twenty-four levels for the A^1^Π state (*ν*′ = 0–23).

Recently, Zhang and Shi^14^ used the icMRCI+Q method to study selected states of the CO molecule. The B^1^Σ^+^ state obtained has one barrier at 1.3 Å, and the potential energy is 6400 cm^−1^ higher than the dissociation limit at this internuclear distance.

For the ground state, Meshkov *et al.*^29^ recently made a semi-empirical function for the ground state permanent dipole moment of CO to construct a new line list. The authors recalculated the intensities of CO isotopologues using new potential energy and dipole moment functions for five vibrational bands within the ground electronic state. They found a deviation in the third and fifth overtones by 1%, and that deviation in the 5–0 band is not significant due to the large uncertainty in the experimental data. Subsequent combined experimental and theoretical work^47,48^ has provided high accuracy intensities for the (3–0) and (7–0) bands.

This article presents a spectroscopic model of the CO molecule's electronic states. We concentrate on highly excited states, where the adiabatic potential energy and transition dipole moment curves are calculated, followed by diabatization, to produce accurate diabatic potential energy and transition dipole moment curves for the B^1^Σ^+^–X^1^Σ^+^ and C^1^Σ^+^–X^1^Σ^+^ transition bands. Synthetic spectra for the A^1^Π–X^1^Σ^+^, B^1^Σ^+^–X^1^Σ^+^, C^1^Σ^+^–X^1^Σ^+^, D′^1^Σ^+^–X^1^Σ^+^ and E^1^Π–X^1^Σ^+^ transitions are computed and compared with the literature.

Additionally, a comparison is made between a diabatic representation with/without diabatic couplings (DCs) and an adiabatic representation without non-adiabatic couplings (NACs), mainly showing an important effect of the diabatic couplings on the line positions. The article is organized as follows: following the introduction given in Section 1, the computational methodology is presented in Section 2. Section 3 is dedicated to the results and discussion, encompassing the electronic structure, diabatization technique, rovibrational calculations, and the impact of the diabatic couplings on the synthetic spectra. The conclusions are given in Section 4.

## 2 Computational methodology

### 2.1 *Ab initio* calculations

The potential energy curves (PECs) and transition dipole moment curves (TDMCs) of the CO molecule were calculated using the MOLPRO 2022.1.2 package,^49^ using the *C*_2v_ group symmetry. The MOLPRO code provides an accurate description of the electronic correlation problem.^49,50^ The calculations of the ground and excited states were investigated using the complete active space self-consistent field (CASSCF) method, followed by an internally contracted MRCI+Q calculation, as done in previous work.^51–53^ One well-know issue with this methodology is obtaining smooth curves (both PECS and TDMCs) as these often show artificial features due to orbital swapping as a function of bondlength.^54^

As done in previous work,^54,55^ an augmented triple-*ζ* correlation-consistent polarized basis (aug-cc-pVTZ)^56–58^ was used for the carbon and oxygen atoms. Only the spd functions were adopted to decrease computational cost and reduce orbital swapping for smoother-shaped curves. Although not optimal, the truncation of the f functions and the neglect of the higher-level (g, *etc.*) functions in basis sets used to describe CO-containing complexes has already been tested and has been proven to have little effect on the main physical properties of the molecules.^59^ Twelve molecular active orbitals were considered, labeled as [6a_1_, 3b_1_, 3b_2_, 0a_2_] in the *C*_2v_ point group, resulting from 6σ and three pairs of π orbitals mainly built from C: 2s, 2p, 3s, 3p and O: 2s, 2p shells. The C 1s and O 1s orbitals are kept doubly occupied in all configurations. In the CAS-SCF calculation, the ten CO valence electrons were distributed into the twelve valence molecular orbitals hence, this active space is referred to as CAS (10, 12). The four inner electrons were placed into the two closed-shell orbitals, which included only two a_1_ symmetry molecular orbitals, corresponding to the 1σ and 2σ molecular orbitals in the CO molecule. There was no change in the total orbital space in the subsequent MRCI calculations.

Using the notation [*A*_1_,*B*_1_,*B*_2_,*A*_2_], the number of states in the irreducible representations of the *C*_2v_ point group considered in the CASSCF calculations is [8,3,3,2] which means 8 *A*_1_, 3 *B*_1_, 3 *B*_2_ and 2 *A*_2_. This [8,3,3,2] states model was used to compute potential energy curves and transition dipole moments for the B^1^Σ^+^–X^1^Σ^+^, C^1^Σ^+^–X^1^Σ^+^, D′^1^Σ^+^–X^1^Σ^+^ and E^1^Π–X^1^Σ^+^ bands. However, a [6,3,3,2] states model was used to calculate potential energy curves and transition dipole moments for the A^1^Π–X^1^Σ^+^ band because the shape of the potential energy curve is slightly different around the minimum for the two CASSCF orbital sets. The choice of six A_1_ states gave more accurate line positions for A^1^Π–X^1^Σ^+^ transitions. Identical energy values for degenerate states were validated for all the calculated potential energy curves.

States with *A*_1_ and *A*_2_ symmetry were only considered in the MCSCF/CASSCF calculations in the region from 0.9 to 1.14 Å, for the singlet states, because of insufficient overlap in the configuration interaction (CI) for the higher states. Excellent agreement was obtained by superimposing the MRCI results from MCSCF/CASSCF calculations that includes only *A*_1_ and *A*_2_ and MCSCF/CASSCF calculations that includes *A*_1_, *B*_1_, *B*_2_, and *A*_2_, in the range from 1.18 to 3.4 Å, which allowed us to use the assumption as a valid extrapolation of the curves outside the 0.9 to 1.14 Å region. Many of the alternative models encountered issues with the active space adopted, such as avoided crossing sometimes moving according to the configuration used and configuration interaction calculations giving inefficient overlap error over the whole range of internuclear distances.

### 2.2 Diabatization

The *ab initio* PECs of CO shown in Fig. 1 and 2 are given in the so-called adiabatic representations. The adiabatic representation consists of a set potential energy curves, in which the electronic Hamiltonian is diagonal, a non-diagonal kinetic energy matrix consisting of non-adiabatic couplings (NACs) and diagonal Born–Oppenheimer corrections (DBOCs). It is common to omit the NAC terms and this has proven to be useful in predicting near equilibrium properties for many molecules.^60^ NACs arise between different electronic states of the same symmetry through action of the nuclear kinetic energy operator on the electronic wavefunctions and correspond to first derivative couplings. The associated PECs in the adiabatic representation are then characterized by avoided crossings near degeneracy,^61^ at which the NAC terms are strongest.^62–65^ In the region of the avoided crossing, the adiabatic PECs can have complex shapes, and the NACs are cusp-like. This is undesirable to treat computationally and represent analytically, especially as such topology can be sensitive to the quality of *ab initio* calculations. Conversely, a diabatic representation of the potential energy curves (as well as associated couplings) exists where NACs vanish simultaneously with the DBOC terms *via* a bond-length-dependent unitary transformation *U*(*r*), and is characterised by a set of PECs that cross at the cost of introducing diabatic couplings (DCs).^66–68^

The diabatizing unitary matrix *U*(*r*) for the coupled two-electronic state system parametrically depends on the NACs through the mixing angle *β*(*r*) and is equivalent to the two-dimensional rotation matrix given by1
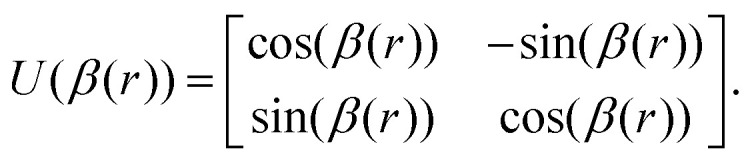


The mixing angle relates the diabatizing transformation to the NAC *ϕ*_12_(*r*) through its integral *via*
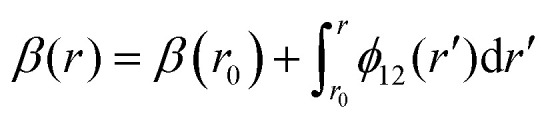
where 
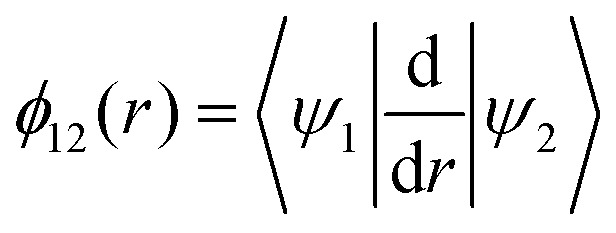
 is the NAC and *β*(*r*_0_) is chosen to ensure physical conditions on the adiabatic–diabatic transformation. |*ψ*_1_〉 represents the adiabatic lower energy electronic wavefunction and |*ψ*_2_〉 represents the adiabatic upper energy electronic wavefunction. The adiabatic representation of PECs for these two states is given by
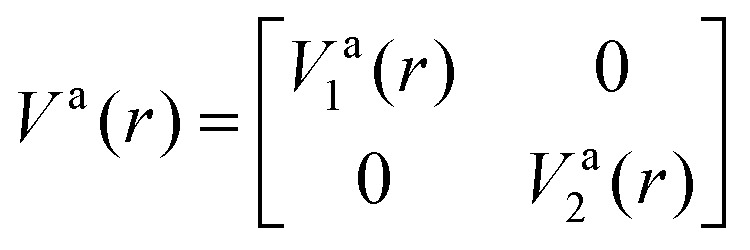
where *V*^a^_1_(*r*) and *V*^a^_2_(*r*) are the adiabatic lower and upper PECs, respectively. The diabatic Hamiltonian is then calculated by applying the unitary matrix to the above adiabatic potential matrix, yielding
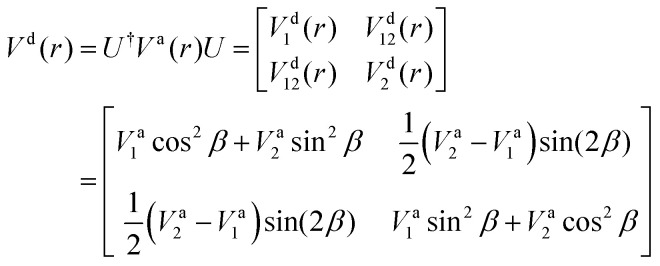


The superscript “d” refers to the diabatic basis and “a” refers to the adiabatic one, while the off-diagonal terms *V*^d^_12_(*r*) are the DCs.

The diabatization method we adopt follows the approach extensively tested by Brady *et al.*^69^ where, instead, the diabatizing transformation and associated NACs are computed *via* a property-based method using the code presented by Brady *et al.*,^70^ as opposed to directly from the NACs. The result being smooth diabatic PECs that are easily parameterised. This method works by optimising a NAC that, when used to transform the *ab initio* adiabatic PECs to the diabatic representation, produces the smoothest diabatic PECs by minimising the following loss function2
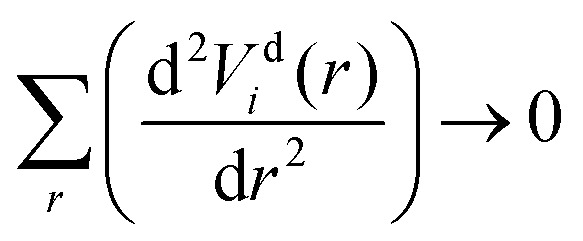


We chose to model the NACs by the combination of a Lorentzian and Laplacian function through the geometric average of their corresponding mixing angles (see Brady *et al.*^70^ and An and Baeck^71^ for details). The Lorentzian function tends to underestimate the NAC at the avoided crossing but overestimates them at large distances from the avoided crossing.^71–73^ Conversely, the Laplacian underestimates the NAC away from the avoided crossing and overestimates the NAC at the avoided crossing. Therefore, their combination ensures the functions’ undesirable properties are mitigated. The Lorentzian NAC curve is given by3
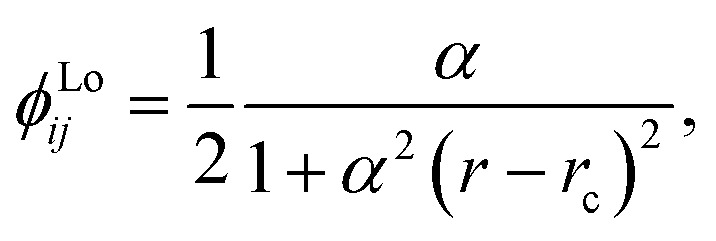
where *r*_c_ is the crossing internuclear distance and *α* is the inverse of the half-width-at-half-maximum (HWHM). The Laplacian NAC curve is given by:4
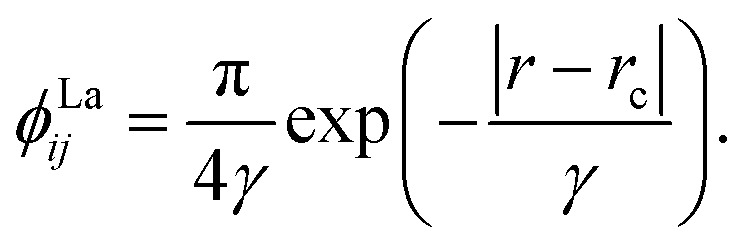
where (*γ*) is a damping constant related to the HWHM.

In this work, we use the diabatic representation for the solution of the nuclear motion Schrödinger equation and calculate spectra of CO with the corresponding PECs and DCs computed using the property-based diabatization technique. More details on the diabatization procedure specific to the investigated CO system are given in Section 3.2. The diabatic curves obtained were used in generating the synthetic spectra discussed later on in Section 3.3.

### 2.3 Rovibrational calculations

Spectral calculations were performed using the Duo variational nuclear-motion program,^74,75^ which treats the rovibronic problem for open shell diatomic molecules by solving numerically the coupled Schrödinger equation. The main output from Duo is a molecular line list. In this paper, Duo is used to calculate line lists for the A^1^Π–X^1^Σ^+^, B^1^Σ^+^–X^1^Σ^+^, C^1^Σ^+^–X^1^Σ^+^, D′^1^Σ^+^–X^1^Σ^+^, and E^1^Π–X^1^Σ^+^ electronic band systems individually. A uniform grid of 501 points was set to solve the coupled Schrödinger equation using the sinc DVR (discrete variable representation) basis set. The calculations are done over a range from 1 to 3.5 Å for A^1^Π–X^1^Σ^+^, from 0.92 to 2 Å for the B^1^Σ^+^–X^1^Σ^+^, from 0.92 to 1.5 Å for C^1^Σ^+^–X^1^Σ^+^, from 0.88 to 1.26 Å for E^1^Π–X^1^Σ^+^, and from 0.75 to 2 Å for D′^1^Σ^+^–X^1^Σ^+^. The maximum energy limit was set to 130 000 cm^−1^ in our model, and the maximum rotational quantum number *J* was set to 110.

The ground state X^1^Σ^+^ and excited A^1^Π state PECs were represented by the Extended Morse Oscillator (EMO) function.^76^ The EMO function is given by the following equation:^74^

5 *V*(*r*) = *T*_e_ + (*A*_e_ − *T*_e_)(1 − exp[−*β*(*r*)(*r* − *r*_e_)])^2^

where,6
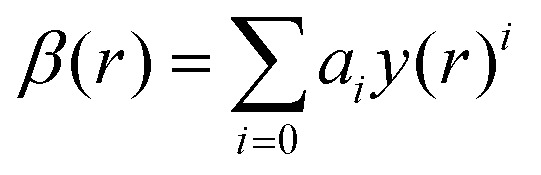
and

7 *y*(*r*) = (*r*^p^ − *r*^p^_e_)/(*r*^p^ + *r*^p^_e_).

*T*_e_ is the minimum electronic energy, *A*_e_ is the asymptotic limit, and *r*_e_ is the equilibrium separation. The initial values of the EMO expansion coefficients were obtained by fitting to the *ab initio* values using the CurveExpert software^77^ before being introduced in Duo in the form of grids. Following the fitting procedure, the PECs were shifted to align with the *T*_e_ and *r*_e_ values taken from Huber and Herzberg.^44^ In more detail, the PEC of A^1^Π was shifted by 0.0105 Å and 27.24 cm^−1^ to assure this alignment. Similar matching of the *T*_e_ and *r*_e_ values were used by Semenov *et al.*^78^ for the PN molecule. For the *ab initio* B^1^Σ^+^ and C^1^Σ^+^ PECs, a similar procedure was followed, where ninth-degree polynomials were used as fitted with ORIGIN.^79^ The ESI† contains the PEC grids (initial *ab initio* data and as introduced in Duo) and proposed fitting parameters.

Absorption spectra for each band were simulated using the code ExoCross.^80^ ExoCross produces spectra and absorption cross-sections at pre-specified temperature using the line list in the ExoMol format^81^ generated by Duo as an input. For each comparison with the experiment, the experimental conditions of temperature and pressure are used as input. The line intensity (absorption coefficient, cm per molecule) is calculated using the following equation:^80^8
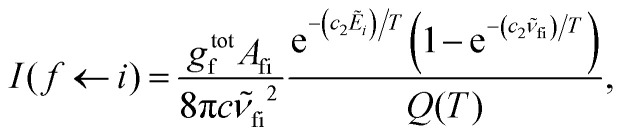
where *ν̃*_fi_ is the transition wavenumber (cm^−1^), *A*_fi_ is the Einstein *A* coefficient (s^−1^), *Ẽ*_*i*_ = *E*_*i*_/*hc* is the energy term value (cm^−1^), *T* is the temperature in K, *c*_2_ is the second radiation constant (*c*_2_ = *hc*/*k*_B_) in cm K, and *Q*(*T*) is the partition function
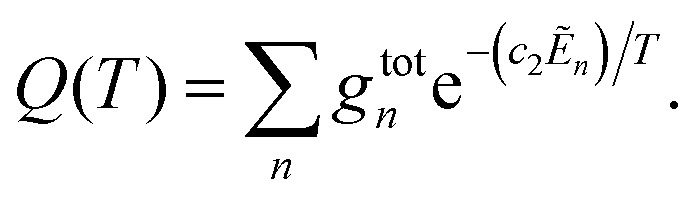


Here, *g*^tot^_*n*_ is the total degeneracy *g*^tot^_*n*_ = *g*^ns^(2*J*_*n*_ + 1) that is a function of the nuclear-spin statistical weight factor (*g*^ns^), which equals 1 for ^12^C^16^O, and the rotational quantum number of the *n*th level (*J*_*n*_).^80^

## 3 Results and discussion

### 3.1 Electronic structure

Fig. 1 and 2 show the adiabatic potential energy curves for singlet and triplet states of the CO molecule, respectively, over a range of internuclear distances (0.95 to 3.5 Å). The asymptotic limit has been corrected to eliminate the numerical/computational error that caused its slight upward shift. A similar case is depicted as numerical noise by Brady *et al.*^70^ in their *ab initio* data for SO. The error resulted in a shift of approximately 0.27 to 0.33% in the corrected asymptote, and it has been rectified in our data. This problem occurs beyond 2.66 Å, a region that was not utilized to generate the synthetic spectrum. The lowest dissociation limits of the calculated low-lying electronic states of the CO molecule have been compared with the combination of atomic term values provided by the National Institute of Standards and Technology (NIST) Atomic Spectra Database,^82^ using the Wigner–Witmer correlation rules,^83^ to confirm that this correction is valid. The relative error for the asymptotic limit of the second singlet states C(^1^D) + O(^1^D) was calculated before and after correction. It was found that the relative error when compared to NIST values, decreased from 9% to 7%.

The singlet states obtained show double-well structures in the adiabatic representation, such as in the case of BD′ and CC′ states. Some valence electronic states such as D′ and C′ have shallow wells due to overcoming the repulsive forces over the attractive ones within the range of the internuclear distances considered.^50^ The other low-lying states mostly have wells, except for the repulsive ^1^Π (IV) state. The depth of the potential energy curve indicates the bond strength and the stability of the molecule in the specified state.^84^

The D′^1^Σ^+^ state almost overlaps with the I^1^Σ^−^ state as portrayed by the similarity of their spectroscopic constants. The difference in the minimum energy between them is approximately 293.8 cm^−1^ at their equilibrium bond length of 1.4 Å (see Table 2 below).

The B^1^Σ^+^ state is described as having the configuration (4σ^2^5σ^1^1π^4^3sσ) while D′^1^Σ^+^ state was given as (4σ^2^5σ^2^1π^3^2π). Most probably, the D′^1^Σ^+^ potential barrier at 2 Å results from an avoided crossing with C′^1^Σ^+^ that converges to the C(^1^D) + O(^1^D) asymptotic limit.^40^

The potential barrier in the BD′ state is calculated by Vázquez *et al.*^40^ to be at *R* = 1.32 Å, which is longer than our value by 0.04 Å and that obtained by Zhang and Shi^14^ using an icMRCI+Q calculation, by 0.02 Å.

Cooper and Kirby^33^ calculated adiabatic potential energy curves using Slater type basis set in MCSCF-CI calculations that are singly excited. The avoided crossing of the BD′ state with the C^1^Σ^+^ state is slightly visible in their calculation at around 1.32 Å. Li *et al.*^21^ conducted an adiabatic calculation for the B^1^Σ^+^ and C^1^Σ^+^ states using MRD-CI. They used non-adiabatic couplings and nuclear kinetic energy corrections to estimate the diabatic effects. The maximum calculated value of the NAC was close to 1.25*a*_0_^−1^ at 1.286 Å where the avoided crossing point is situated, which is close to the avoided crossing position value we obtain in our calculations.

Li *et al.*^21^ reported that the BD′ state consists of the B^1^Σ^+^ Rydberg state and D′^1^Σ^+^ valence state character (π^3^π*^3^),^21^ while C^1^Σ^+^ state is mixed between the Rydberg (2sσ) and (3pσ) and D′^1^Σ^+^ valence character. The same authors conclude that the BD′ state has an observed second energy well, which supports three vibrational levels,^21^ similar to the bound region of the D′^1^Σ^+^ state obtained in the present study.

The second adiabatic ^1^Π state has two avoided crossings. The first one is between E^1^Π and E′^1^Π, and the second one is between E′^1^Π and the unbound ^1^Π(IV) state^40,42^ that we obtain in our calculations.

The term values *T*_v*J*_ of a vibrating rotator can be expressed as a function of the minimum electronic energy *T*_e_, the vibrational constant *ω*_e_, the first, second and third order anharmonicity constants *ω*_e_*x*_e_, *ω*_e_*y*_e_, and *ω*_e_*z*_e_, the vibrationally averaged rotational constant *B*_v_, and the vibrationally averaged centrifugal distortion constant, *D*_v_ through the equation:^83^9
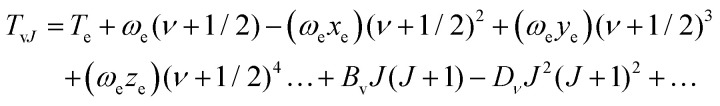
where *B*_v_ can be expressed as a function of the equilibrium rotational constant, *B*_e_, and the first, second, and third-order vibration–rotation interaction constants, *α*_e_, *γ*_e_ and *δ*_e_ as:

10 *B*_*ν*_ = *B*_e_ − *α*_e_(*ν* + 1/2) + *γ*_e_(*ν* + 1/2)^2^ + *δ*_e_(*ν* + 1/2)^3^ + …

where

11 *B*_e_ = *h*/(8π^2^*cI*_e_)

Here, *h* is Planck's constant, *I*_e_ is the moment of inertia of the molecule at the equilibrium bond distance, and *c* is the speed of light. Similarly, *D*_v_ can be expressed in terms of the equilibrium centrifugal distortion constant *D*_e_, and the first, second and third order vibration–rotation interaction constants *β*_e_, 
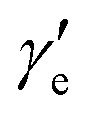
 and 
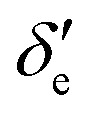
 as:12
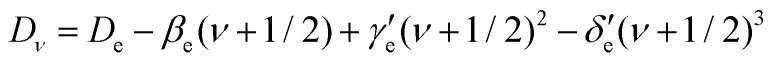
where

13 *D*_e_ = 4*B*_e_^3^/*ω*_e_

The spectroscopic constants of the electronic states of CO molecule obtained in this work were calculated using a polynomial fitting program. Their comparison with theoretical/experimental data from the literature is presented in Table 2. Our results show excellent agreement with the available results for most calculated states.

The calculated equilibrium internuclear distance *R*_e_ and rotational constant *B*_e_ for the ground state X^1^Σ^+^ of the CO molecule agree well with the ref. 33, 44, 45 and 85–88, with relative error of 0.07% ≤ Δ*R*_e_/*R*_e_ ≤ 0.86% and 0.97% ≤ Δ*B*_e_/*B*_e_ ≤ 1.75%. Also, the A^1^Π state has an equilibrium internuclear distance of 1.24 Å and *T*_e_ value of 65 036.27 cm^−1^ which corresponds to a relative error of 0.14% ≤ Δ*R*_e_/*R*_e_ ≤ 0.87% and 0.06% ≤ Δ*T*_e_/*T*_e_ ≤ 2.73%, with respect to the experimental values reported by Herzberg,^44^ O’Neil and Schaefer,^89^ as well as other theoretical studies by Vázquez *et al.*,^40^ Cooper and Kirby,^33^ Lu *et al.*,^45^ Chantranupong *et al.*,^85^ Shi *et al.*,^86^ Majumder *et al.*^90^

The adiabatic electronic excitation threshold (*T*_e_) of the Hopfield–Birge band states B^1^Σ^+^ and C^1^Σ^+^ is calculated to be 87 964.43 and 92 566.1 cm^−1^, respectively. The relative error for the B^1^Σ^+^ is 0.77% ≤ Δ*T*_e_/*T*_e_ ≤ 1.190% and 0.1% ≤ Δ*T*_e_/*T*_e_ ≤ 0.71% for the C^1^Σ^+^ state when compared to Huber and Herzberg,^44^ Vázquez *et al.*,^40^ Eidelsberg *et al.*,^91^ Eidelsberg and Rostas,^92^ respectively.

The valence state D′^1^Σ^+^ has a calculated equilibrium internuclear distance of 1.650 Å and *T*_e_ value of 87 338.05 cm^−1^ which corresponds to a relative error of 2.44% ≤ Δ*r*_e_/*r*_e_ ≤ 4.45% and −0.65% ≤ Δ*T*_e_/*T*_e_ ≤ −2.35%, with respect to the experimental and theoretical values reported by Wolk and Rich,^37^ Vázquez *et al.*,^40^ respectively. The minimum energy of the E^1^Π state is 94 408.3 cm^−1^ at 1.130 Å, which corresponds to a relative error of 0.82% ≤ Δ*r*_e_/*r*_e_ ≤ 1.44% and 1.62% ≤ Δ*T*_e_/*T*_e_ ≤ 1.9%, with respect to the experimental and theoretical values reported by Vázquez *et al.*,^40^ Huber and Herzberg,^44^ respectively.

**Fig. 3:**
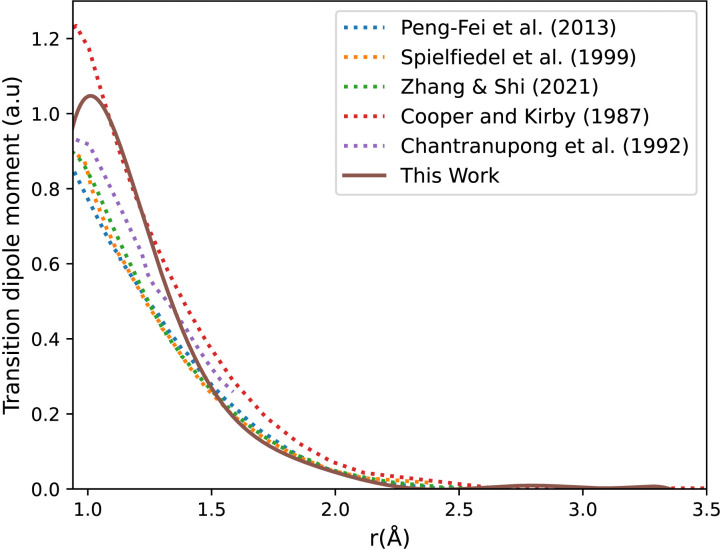
CO A^1^Π–X^1^Σ^+^ transition dipole moment curves: ours compared to literature curves.^35,45,85,88^

Fig. 3 shows our A^1^Π–X^1^Σ^+^ calculated transition dipole moment that is fitted to a ninth-degree polynomial with a standard deviation of the residuals 0.01 a.u. It shows good agreement with the curves extracted from literature.^14,35,45,85,88^

**Fig. 4:**
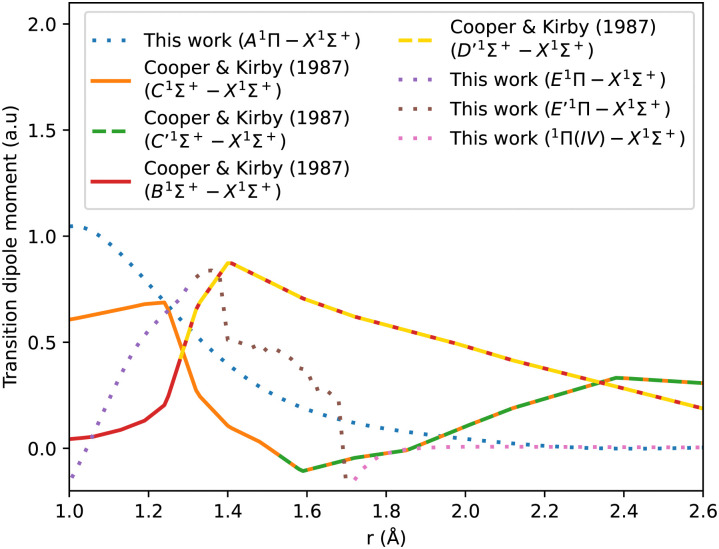
Transition dipole moment curves for A^1^Π–X^1^Σ^+^, B^1^Σ^+^–X^1^Σ^+^, C^1^Σ^+^–X^1^Σ^+^, E^1^Π–X^1^Σ^+^, E′^1^Π–X^1^Σ^+^, and ^1^Π(IV)–X^1^Σ^+^.

The adiabatic transition dipole moment curves (TDMCs) used in the calculations are shown in Fig. 4. The E^1^Π–X^1^Σ^+^ TDMC is calculated in the adiabatic representation (its diabatic representation is out of scope of this work), so only the portion before the avoided crossing of the transition dipole moment is considered for this state.

As can be seen in Fig. 1, the adiabatic ^1^Π states E^1^Π, E′^1^Π and ^1^Π(IV) undergo avoided crossings between each other. Fig. 4 displays their TDMs for transitions with the X^1^Σ^+^ state. Different regions of the TDM are assigned for each state by different colours.

The electronic structure *ab initio* curves presented by Kirby and Cooper^35^ appear to closely resemble ours, with a slight difference in the region around the avoided crossing. The shift observed in our curves beyond 2.66 Å is typical in the excited states of CO as MOLPRO calculations become particularly noisy in this region due to orbital swapping.^93^

The diabatic transition dipole moment curves for the B^1^Σ^+^–X^1^Σ^+^ and C^1^Σ^+^–X^1^Σ^+^ systems were constructed from Kirby and Cooper's adiabatic transition dipole moment curves. In fact, the crossing point between the B^1^Σ^+^–X^1^Σ^+^ and C^1^Σ^+^–X^1^Σ^+^ TDM's in our data occurs at 1.40 Å, whereas in Kirby and Cooper^35^ data, it is at 1.28 Å. At the same time, Kirby and Cooper^35^'s TDMs are smoother and show more consistency in the range investigated. Consequently, the diabatization procedure followed resulted in more conclusive results using their calculated TDMs than when our own dipole curves were used. All other transition dipole moment curves used in this paper are our calculated curves. It is worth mentioning that Kirby and Cooper^35^ potential energy curves are also shifted with respect to their transition dipole moment curves’ crossing point by 0.04 Å.

### 3.2 Diabatized potential energy and transition dipole moment curves

As stated in the introduction, the 2^1^Σ^+^ (or BD′) state which is constituted by the Rydberg B^1^Σ^+^ and valence D′^1^Σ^+^ states, makes an avoided crossing with the upper 3^1^Σ^+^ (or CC′) state in the C^1^Σ^+^ region of the curve, close to 1.28 Å. The CC′ state also makes another avoided crossing with the higher Σ^+^(IV) state. There is also an avoided crossing at about 2 Å between the D′^1^Σ^+^ and C′^1^Σ^+^ states but it is not considered here as it is distant from the 1.28 Å region. In this work, we simplify the complexity of the 3-level system constituted of the 2^1^Σ^+^, 3^1^Σ^+^ and Σ^+^(IV) states, and instead aim to produce a physically meaningful model of CO that gives a good spectroscopic model using initially a 2-level system which would be sequentially related to the remaining state, leading to a 3-levels system. We will first concentrate on the avoided crossings between the BD′ and C^1^Σ^+^ states (Section 3.2.1), then use the obtained results to make deductions about the avoided crossing between the C^1^Σ^+^ and Σ^+^(IV) states (Section 3.2.2).

#### 3.2.1 Diabatization of the BD′–C^1^Σ^+^ system

**Fig. 5:**
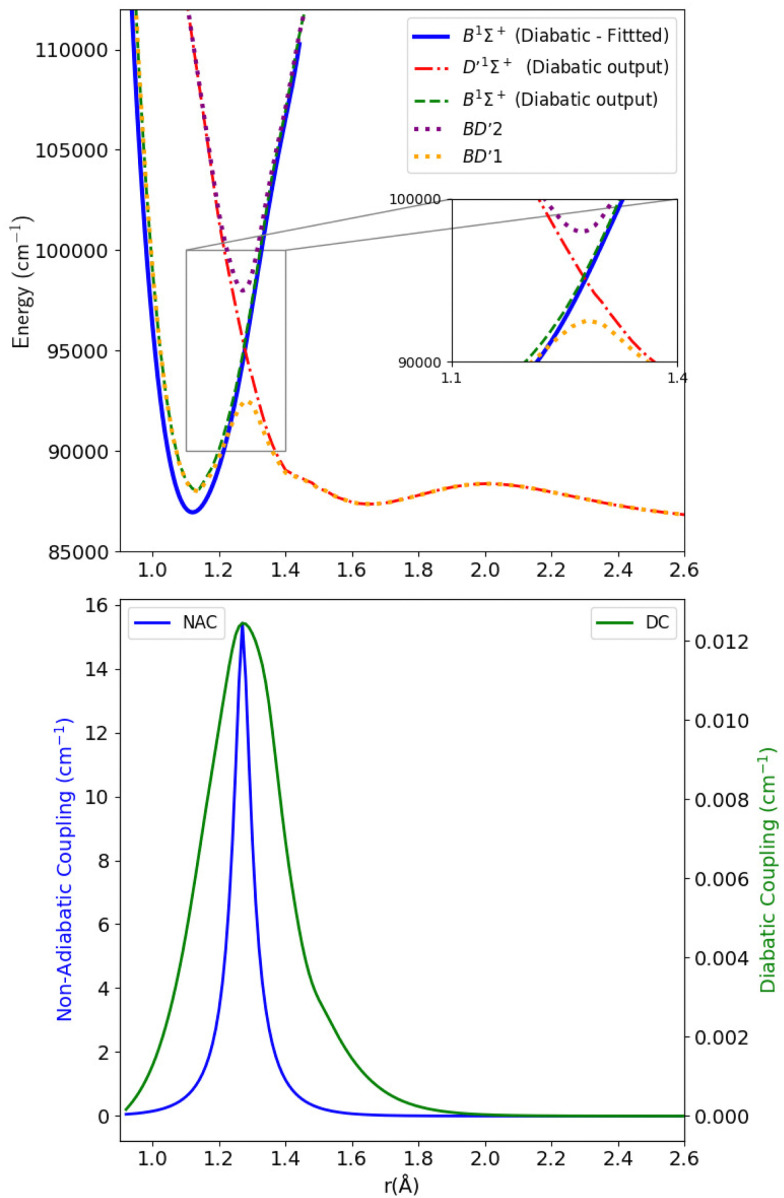
Upper panel: Adiabatic and diabatic potential energy curves for the BD′ system. Lower panel: The NACs and DCs of the BD′ system.

Fig. 5, shows the adiabatic (BD′) and diabatic B^1^Σ^+^ and D′^1^Σ^+^ potential energy curves of CO in the first subplot, and the NACs and the DCs in the second subplot. BD′1 and BD′2 indicate the actual lower and upper adiabatic states, respectively. This notation was proposed by Tchang-Brillet *et al.*^39^ The two corresponding dotted curves have an avoided crossing at 1.28 Å, of which only a small protrusion appears, as shown in the inset of Fig. 1. Given this incompleteness of the avoided crossing, and to go through the diabatization process, the purely adiabatic BD′1 curve obtained in the *ab initio* calculations is considered as the lower state, while BD′2, considered as the higher quasi-adiabatic state, is constructed/completed by fitting analytically the *ab initio* data points of the C^1^Σ^+^ state from 1.28 to 1.48 Å with a ninth-degree polynomial which extrapolates the left side profile of this upper state without affecting the position of the avoided crossing. This fitting was crucial for constituting the upper quasi-state, thereby facilitating the diabatization procedure. The fitting quality was verified by taking the second derivative of the lower and the upper curves using the OriginPro software.^79^ The dashed lines in Fig. 5, are the calculated diabatic curves for the B^1^Σ^+^ and D′ states after using the property-based diabatization code by Brady *et al.*^70^

The thick blue solid line represents the B^1^Σ^+^ state curve after fitting to the experimental *T*_e_ and *r*_e_ spectroscopic constants values. The NACs for the BD′ system are very small, with a maximum value of 15.418 cm^−1^ at the crossing point (Fig. 5). The small value of NACs indicates weak interaction between the B^1^Σ^+^ and D′^1^Σ^+^ states at the crossing point.^70^ In addition, the maximum value of the diabatic couplings is minimal (0.013 cm^−1^). These were smoothed, before being introduced to Duo, with the Savitzky–Golay digital signal processing method with a value of standard deviation of residuals *σ*_r_ = 0.0000313 cm^−1^.

**Fig. 6:**
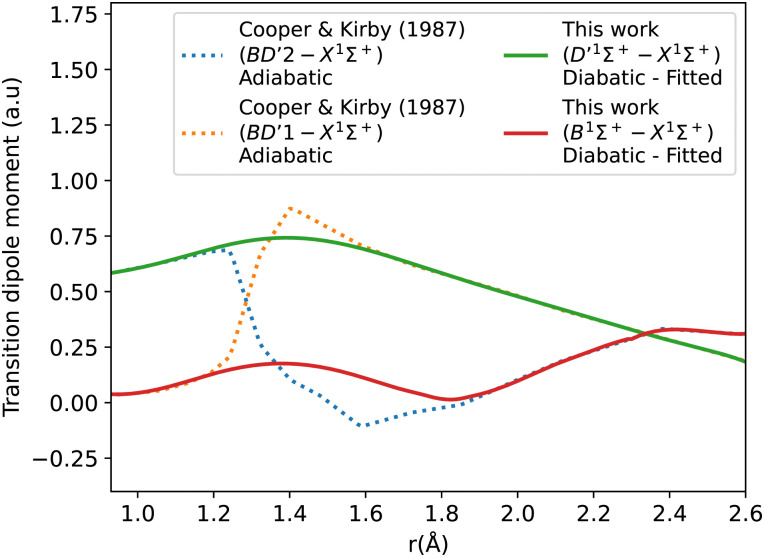
Adiabatic and diabatic B^1^Σ^+^–X^1^Σ^+^ transition dipole moment curves.

Fig. 6 shows the adiabatic and diabatic transition dipole moment curves used in the B^1^Σ^+^–X^1^Σ^+^ transition calculations. The dotted curves^35^ are adiabatic TDMs, corresponding to transitions to the ground state, for which the adiabatic C^1^Σ^+^ state fitted and extrapolated part is denoted as BD′2, and the adiabatic B^1^Σ^+^ state is denoted as BD′1. The solid lines represent the B^1^Σ^+^–X^1^Σ^+^ and the D′^1^Σ^+^–X^1^Σ^+^ diabatic TDMs.

The polarity in the diabatic B^1^Σ^+^–X^1^Σ^+^ curve was corrected by flipping its sign beyond 1.87 Å, allowing it to follow the polarity of the BD′2–X^1^Σ^+^ curve. This adjustment is akin to inducing two flips to account for the simplification of the 3-level system to a 2-level system. Both diabats are smoothed after the diabatization procedure because the ones obtained directly from the diabatization process exhibit a jump at the avoided crossing, also possibly due to numerical errors arising from the simplification of the 3-level system to a 2-level system. The B^1^Σ^+^–X^1^Σ^+^ TDM used in the calculation is smoothed using a Savitzky–Golay filter and cubic spline with 0.0037 a.u. value of residuals standard deviation (*σ*_r_). The accuracy of the shape of the transition dipole was tested by comparing the intensity of the B–X (1–0) to B–X (0–0) bands. Our calculated relative intensity (RI) is 0.065, which agrees well with the experimental values of Imhof *et al.*^94^ (RI = 0.066), and Chan *et al.*^95^ (RI = 0.067). Furthermore, the quasi C^1^Σ^+^–X^1^Σ^+^ TDM not used in the calculations is also smoothed using an eighth-degree polynomial with *σ* = 0.033 a.u.

The diabatization smooths out the curves and eliminates the steep gradient caused by the avoided crossing in the adiabatic representation. The derivative of the dipole moment with respect to internuclear distance (*r*) affects the vibronic intensities. So, having diabatic curves will reduce the inaccuracy and errors in the spectral properties of the molecule because there is no sudden jump in the diabatic curves as in the case of the adiabatic ones.^74^

#### 3.2.2 Diabatization of the C^1^Σ^+^–^1^Σ^+^(IV) system

**Fig. 7:**
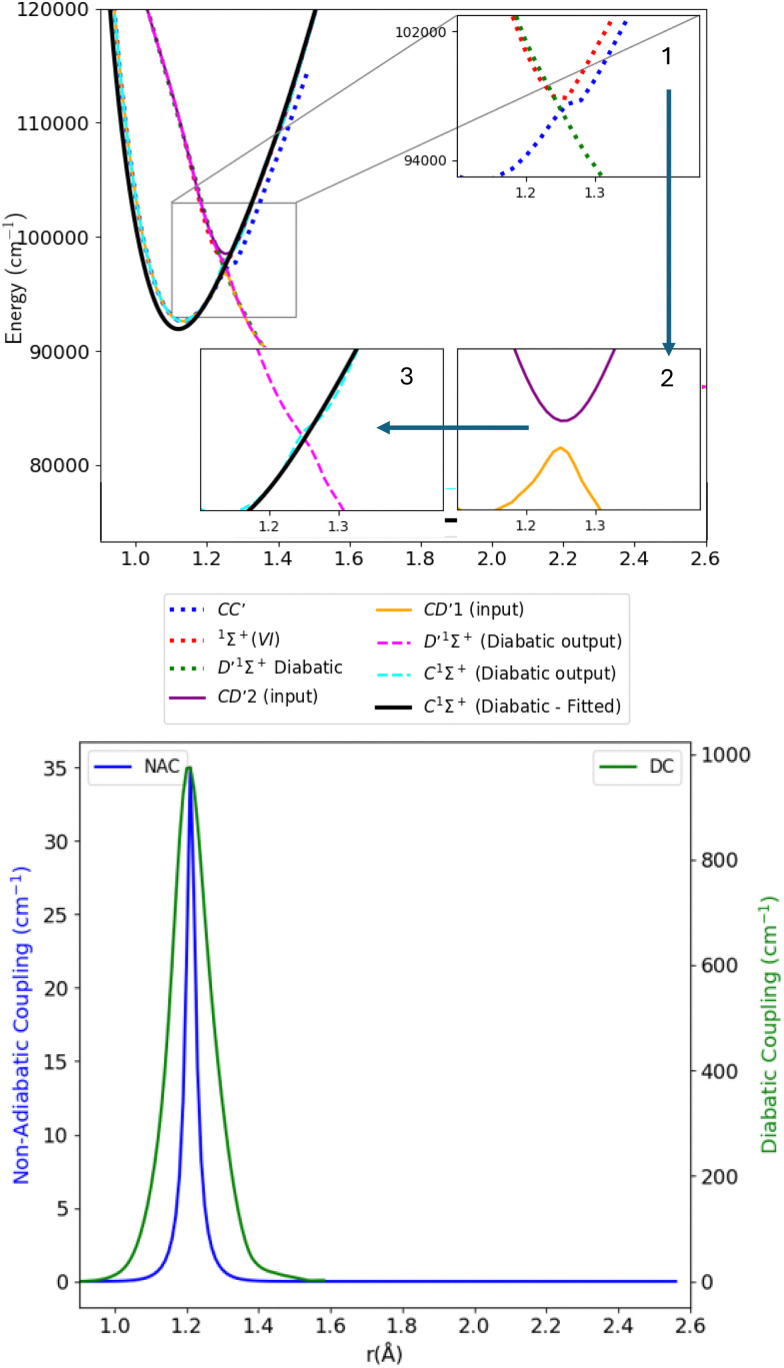
Upper panel: Adiabatic and diabatic potential energy curves for CD′ system. Lower panel: The NACs and DCs of the CD′ system.

Fig. 7 shows the adiabatic and diabatic curves used to diabatize the C^1^Σ^+^ state. Through this process, we treat the avoided crossing between the C^1^Σ^+^ and ^1^Σ^+^(IV) states (shown as the pink and purple curves in Fig. 1) similarly to the BD′–C^1^Σ^+^ avoided crossing discussed in the previous sub-section. In this case also, in order to analyze the system, quasi-states were constructed to represent intermediate adiabatic lower and upper states, denoted as CD′1 and CD′2. In more detail, the CD′1 state is constructed using the adiabatic C^1^Σ^+^ state (lower blue dotted curve of inset 1 in Fig. 7 as left portion) and the diabatic D′^1^Σ^+^ state obtained from treating the BD′ system (lower green dotted curve of inset 1 in Fig. 7 as right portion). The avoided crossing was smoothed using a ninth-degree polynomial fitting between 1.18 to 1.28 Å while maintaining the maximum of the potential barrier at the correct crossing position at 1.25 Å (lower yellow curve of inset 2 in Fig. 7). The upper state CD′2 was formed using the diabatic continuation of the D′^1^Σ^+^ state from 1 to 1.24 Å (upper green dotted curve of inset 1 in Fig. 7 as left portion) and the ^1^Σ^+^(VI) state from 1.26 to 1.4 Å (upper red dotted curve of inset 1 in Fig. 7 as right portion). Similarly, the upper state was fitted using a ninth-degree polynomial over the whole range of the upper state while maintaining the correct minimum energy position at 1.25 Å (upper purple curve of inset 2 in Fig. 7). The two curves constructed (CD′1 and CD′2) were used as input to the Brady *et al.*^70^ code to obtain the diabatic C^1^Σ^+^ and D′^1^Σ^+^ curves. The two diabatic D′^1^Σ^+^ curves, obtained from treating the BD′ and CD′ systems, overlap as expected, and the C^1^Σ^+^ state was fitted similarly as the B^1^Σ^+^ state to the experimental *T*_e_ and *r*_e_ spectroscopic constants values (black curve in inset 3 of Fig. 7). In this case, the maximum value of the NACs is 35.019 cm^−1^. The relatively high value of the NACs indicates a stronger interaction or coupling between the C^1^Σ^+^ and D′^1^Σ^+^ states at the crossing point compared to the BD′ system.^70^ It also indicates that state crossing is widely avoided.^96^ Furthermore, the maximum value of the diabatic couplings (974.543 cm^−1^) is much higher than in the BD′ system, which suggests a higher possibility of energy or electron transfer without requiring any nuclear movement.^97^ As explained, the quasi-states CD′1 and CD′2 were obtained by “stitching” together the upper and lower *ab initio* curves to represent well the avoided crossing system. This procedure leads to PECs and TDM curves that follow well the diabatic trend; however, this representation comes with some disadvantages, mainly in terms of consistancy. For example, there was an asymmetric protuberance in a localised region (1.21 to 1.28 Å) of the CD′ system DCs (Fig. S3, Section S4 in the ESI†). Its source is a protrusion in the C^1^Σ^+^ state, obtained after diabatization, probably resulting from our use of the diabatic D′^1^Σ^+^ curve (calculated in the BD′ system) to estimate the shape of the lower CD′1 and upper CD′2 curves. To solve this problem, the portions of the DC curves outer to the attained region were fitted using the Univariate Spline interpolation method, before incorporating them in Duo to perform the rovibrational calculations. In this case, *σ*_r_ = 6.98 cm^−1^. As stated above, the DCs of the CD′ system reach a maximum value of about 975 cm^−1^. To confirm these results, a direct fit was also made for the same region with a Pearson VII mathematical function having *σ*_r_ = 4.72 cm^−1^, and identical ro-vibrational results were obtained.

Table 1 provides values for the optimized NAC parameters *α*, *γ*, *r*_c_ used to diabatize the BD′ and CD′ systems, which are visualised in Fig. 5 and 7.

**Table 1:** Optimised parameters *α* (inverse Lorentzian HWHM), *γ* (Laplacian damping parameter), and *r*_c_ (avoided crossing) for the NACs used to diabatize the BD′ and CD′ systems

System	*α*	*γ*	*r*_c_ (Å)
BD′	29.937	0.047	1.275
CD′	72.458	0.019	1.246

**Table 2:** Spectroscopic constants of the calculated states of ^12^C^16^O in comparison with literature. RE% is the relative error between the calculated value in this paper and the corresponding reference value. “*” indicates experimental data; values given between square brackets are for *T*_0_, *r*_0_, and Δ*G*_0_ values instead of *T*_e_, *r*_e_, and *ω*_e_

Ref.	State	*r*_e_ (Å)	*r*_e_ |RE%|	*ω*_e_ (cm^−1^)	*ω*_e_ |RE%|	*ω*_e_ *x*_e_ (cm^−1^)	*ω*_e_ *x*_e_ |RE%|	*B*_e_ (cm^−1^)	*B*_e_ |RE%|	*T*_e_ (cm^−1^)	*T*_e_ |RE%|
This work	X^1^Σ^+^	1.14		2125.45		12.29		1.90		0	
44*	X^1^Σ^+^	1.13	0.86	2169.81	2.04	13.29	7.53	1.93	1.75	0	
45	X^1^Σ^+^	1.13	0.47	2141.70	0.76	12.34	0.43	1.92	0.97	0	
87	X^1^Σ^+^	1.13	0.36	2000	6.27					0	
33	X^1^Σ^+^	1.14	0.07	2151.60	1.22	12.90	4.75			0	
85	X^1^Σ^+^	1.13	0.67	2178	2.41	13.00	5.48			0	
88	X^1^Σ^+^	1.13	0.72	2170	2.05	13.90	11.60			0	
86	X^1^Σ^+^	1.13	0.83	2169.47	2.03	13.74	10.57	1.93	1.69	0	
89	X^1^Σ^+^	1.24	8.22	1854	14.64	13.88	11.47	1.61	17.85	0	
This work	a^3^Π	1.22		1729.45		21.24		1.66		48 247.09	
98*	a^3^Π	1.21	0.89	1738.26	0.51	14.25	49.03			48 686.70	0.90
45	a^3^Π	1.21	0.49	1743.41	0.80	14.97	41.86	1.68	1.03	48 796.40	1.13
86	a^3^Π	1.25	2.74	1748.12	1.07	14.39	47.58	1.69	1.81	48 650	0.83
89	a^3^Π	1.33	8.54	1488	16.23	17.80	19.31	1.39	19.47	42 102.10	14.60
89*	a^3^Π	1.21	0.53	1743	0.78	14.50	46.46	1.69	1.74	48 715.90	0.96
This work	a′^3^Σ^+^	1.36		1177.20		29.61		1.33		54 371.28	
44*	a′^3^Σ^+^	1.35	0.59	1228.60	4.18	10.47	182.84	1.34	1.14	55 825.40	2.60
45	a′^3^Σ^+^	1.36	0.19	1214.10	3.04	9.27	219.56	1.33	0.21	55 566.27	2.15
87	a′^3^Σ^+^	1.35	1.13	1240	5.06					55 540	2.10
89	a′^3^Σ^+^	1.48	8.09	1147	2.63	10.60	179.31	1.18	12.65	40 811.60	33.23
89*	a′^3^Σ^+^	1.35	0.76	1231	4.37	11	169.16	1.35	1.54	55 813.50	2.58
This work	d^3^Δ	1.38		1075.77		28.45		1.30		60 226.69	
44*	d^3^Δ	1.37	0.47	1171.94	8.21	10.64	167.52	1.31	0.84	61 120.10	1.46
45	d^3^Δ	1.37	0.15	1158.64	7.15	9.09	212.92	1.30	0.02	60 881.80	1.08
86	d^3^Δ	1.37	0.49	1175	8.44	10.75	164.76	1.31	0.87	61 147.60	1.51
89	d^3^Δ	1.50	8.26	1107	2.82	11.20	154.02	1.09	19.24	48 877.20	23.22
89*	d^3^Δ	1.37	0.44	1153	6.70	7.20	295.15	1.31	0.78	61 136.80	1.49
This work	e^3^Σ^−^	1.40		1027.51		9.66		1.26		63 375.71	
44*	e^3^Σ^−^	1.38	1.07	1117.70	8.07	10.69	9.63	1.28	2.08	64 230.20	1.33
89	e^3^Σ^−^	1.51	7.36	1062	3.25	12.20	20.85	1.07	17.47	51 458.10	23.16
89*	e^3^Σ^−^	1.38	1.36	1114	7.76	9.60	0.59	1.28	1.80	64 201.70	1.29
90	e^3^Σ^−^	1.39	0.63	1106.60	7.15	9.62	0.38			63 747.90	0.58
This work	I^1^Σ^−^	1.40		1041.22		53.63		1.25		64 366.6	
44*	I^1^Σ^−^	1.39	0.62	1092.22	4.67	10.70		1.27	1.33	65 084.40	1.10
45	I^1^Σ^−^	1.40	0.16	1058.71	1.65	9.79		1.25	0.28	65 644.26	1.95
86	I^1^Σ^−^	1.39	0.63	1094.75	4.89	10.71		1.27	1.34	65 087.21	1.11
89	I^1^Σ^−^	1.55	9.70	955	9.03	14.90	259.93	1.03	21.71	53 716.50	19.83
89	I^1^Σ^−^	1.39	0.70	1092	4.65	10.80	396.57	1.27	1.29	65 088.90	1.11
This work	D^1^Δ	1.40		1107.71		29.92		1.26		64 660.4	
44*	D^1^Δ	1.40	0	1094	1.25	10.20	193.37	1.26	0.15	65 928	1.92
89	D^1^Δ	1.56	10.32	905	22.40	12.40	141.32	1.01	24.27	54 442.40	18.77
89*	D^1^Δ	1.40	0.07	1080	2.57	10	199.24	1.26	0.39	65 976.10	1.99
This work	A^1^Π	1.24		1497.22		15.12		1.59		65 036.27	
40	A^1^Π	1.24	0.59	1461.20	2.47	4.40	243.70			64 755.70	0.43
44*	A^1^Π	1.24	0.73	1518.20	1.38	19.40	22.05	1.61	1.54	65 075.70	0.06
45	A^1^Π	1.24	0.29	1505.20	0.53	17.30	12.58	1.60	0.64	65 125.50	0.14
33	A^1^Π	1.25	0.41	1475	1.51	18.90	19.98			66 863.30	2.73
85	A^1^Π	1.24	0.14	1496	0.08	18.10	16.45			65 653.50	0.94
88	A^1^Π	1.23	0.87	1514	1.11	18	15.98			65 492.20	0.70
86	A^1^Π	1.23	0.77	1523.75	1.74	19.20	21.23	1.77	10.14	65 116.30	0.12
89	A^1^Π	1.36	8.50	1357	10.33	22.80	33.67	1.33	19.30	57 104	13.89
89*	A^1^Π	1.24	0.35	1516	1.24	17.30	12.58	1.61	1.45	65 072.80	0.06
90	A^1^Π	1.24	0.51	1509.50	0.81	17.50	13.58			65 076.40	0.06
This work	b^3^Σ^+^	1.11		3163.71		228.22		1.98		82 912.24	
44*	b^3^Σ^+^	1.11	0.03	2199.30	43.85			1.99	0.09	83 814	1.08
This work	^3^Σ^+^(III)	2.70		877.58		0		0.34		86 774.02	
This work	D′^1^Σ^+^	1.65		551.77		30.68		0.90		87 338.05	
91* 92*	D′^1^Σ^+^	1.58	4.45	651.40	15.29	20.40	50.39			89 438.40	2.35
40	D′^1^Σ^+^	1.61	2.44	681.70	19.06	13.40	128.96			87 912	0.65
37*	D′^1^Σ^+^	1.58	4.45	651.40	15.29	20.40	50.39	0.98		89 438.40	2.35
This work	B^1^Σ^+^	1.13		2152.16		62.35		1.92		87 964.43	
91* 92*	B^1^Σ^+^	1.12	0.98	2161.70	0.44	39.80	56.66			86 926.90	1.19
40	B^1^Σ^+^	1.12	0.60	2093	2.83	15.70	297.14			87 292.80	0.77
44*	B^1^Σ^+^	1.12	0.98	2112.70	1.87	15.20	310.21	1.96	2.02	86 945.20	1.17
This work	k^3^Π	1.41		652.31						89 916.37	
40	k^3^Π	1.33	5.60	[882.7]						90 491.40	0.64
99	k^3^Π	1.38	2.08	805.10	18.98	−2.85	1514.73			91 012.20	1.20
This work	J^3^Σ^+^	1.13		2101.30		23.34		1.91		90 837.12	
44*	J^3^Σ^+^	1.14	0.89	2166	2.99	15	55.6	1.88	1.63	90 975	0.15
This work	C^1^Σ^+^	1.13		2119.10		35.63		1.92		92 566.1	
91* 92*	C^1^Σ^+^	[1.1248]		2189	3.19	17.33	105.61			91 914	0.71
40	C^1^Σ^+^	1.12	0.82	2183.12	2.93	16.18	120.23			92 659.80	0.10
44*	C^1^Σ^+^	1.12	0.78	2175.90	2.61	14.70	142.40	1.95		91 916.50	0.71
This work	E^1^Π	1.13		2121.27		30.97		1.92		94 408.3	
44*	E^1^Π	1.12	1.44	2153	1.47	42	26.26	1.98	2.88	92 903	1.62
40	E^1^Π	1.12	0.82	[2127.6]						92 649.40	1.90
100*	E^1^Π	[1.1221]		[2152.9]						[92 929.9]	
This work	c^3^Π	1.13		2160.73		9.62		1.94		93 131.07	
101	c^3^Π	[1.1203]	[2190]		[2190]				[92 076.9]		
40	c^3^Π	1.12	0.82	[1948.9]						91 948.50	1.29
This work	^3^Π(IV)	2.06		517.14		24.02		0.58		95 043.20	
This work	^3^Π(V)	1.19		2607.62		53.10		1.74		96 463.14	
This work	^1^Σ^+^(VI)	1.25		2927.28		64.51		1.57		98 375.29	
This work	^3^Π(VI)	1.38		636.02		18 208.16		1.26		99 171.56	
This work	E′^1^Π	1.41		1458.11		72.31		1.23		99 636.64	
40	E′^1^Π	1.31	7.74	[750]						98 487.70	1.17
This work	C′^1^Σ^+^	1.98		647.96		7.81		0.63		103 185.29	
40	C′^1^Σ^+^	1.94	2.31	709.30	8.65	46.70	83.27			104 127.30	0.90
This work	^1^Π(V)	1.25		1525.60		10.71		1.56		104 403.73	
This work	^3^Δ(II)	1.27		2308.40		307.72		1.52		111 628.07	
This work	^3^Σ^+^(V)	1.28		2736.69		399.45		1.51		112 213.48	
This work	^1^Σ^+^(VIII)	1.26		1304.58		35.49		1.54		112 784.96	
This work	^1^Δ(II)	2.45		329.40		10.34		0.41		113 112.14	
This work	^1^Φ(I)	2.60		1078.95		567.06		0.36		114 130.49	
This work	^1^Σ^+^(IX)	1.24		1175.79		36.48		1.61		114 295.53	

It might be useful to shift the *T*_e_ value at the avoided crossings before the diabatization to improve the shape of the spectra; we leave this as a potential avenue for future work.

**Fig. 8:**
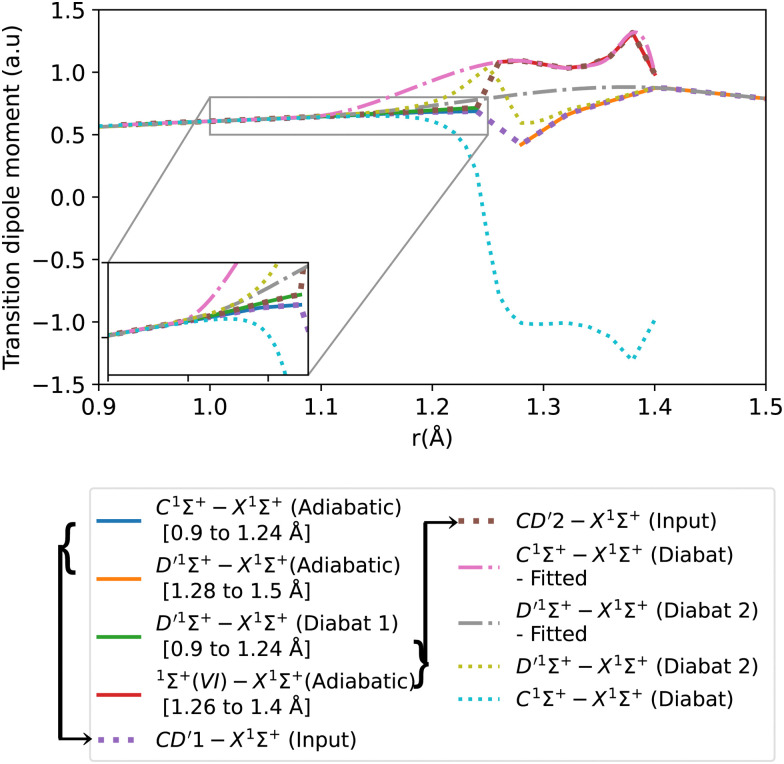
Adiabatic and diabatic C^1^Σ^+^–X^1^Σ^+^ transition dipole moment curves. D′^1^Σ^+^–X^1^Σ^+^ (Diabat 1) denotes the D′^1^Σ^+^–X^1^Σ^+^ diabatic curve obtained from treating BD′ system, and D′^1^Σ^+^–X^1^Σ^+^ (Diabat 2) denotes the D′^1^Σ^+^–X^1^Σ^+^ diabatic curve obtained from treating CD′ system.

The transition dipole moment curves were constructed similarly to the potential energy curves (Fig. 8). The CD′1–X^1^Σ^+^ TDM associated with the lower diabat, is constructed using the adiabatic C^1^Σ^+^–X^1^Σ^+^ TDM from 0.9 to 1.24 Å, and from the adiabatic D′^1^Σ^+^–X^1^Σ^+^ TDM from 1.28 to 1.5 Å. Similarly, the CD′2–X^1^Σ^+^ TDM associated with the upper diabat, is constructed using the first diabatic D′^1^Σ^+^–X^1^Σ^+^ TDM from 0.9 to 1.24 Å and from the adiabatic ^1^Σ^+^(VI)–X^1^Σ^+^ TDM from 1.26 to 1.4 Å. The first diabatic D′^1^Σ^+^–X^1^Σ^+^ TDM is obtained from treating the BD′ system and denoted Diabat 1.

After the diabatization process, two diabatic TDMCs are obtained, C^1^Σ^+^–X^1^Σ^+^ and D′^1^Σ^+^–X^1^Σ^+^. The diabatized CD′ system is denoted Diabat 2. Here, the D′^1^Σ^+^ state was used as a tool to facilitate the diabatization process. The diabatic C^1^Σ^+^–X^1^Σ^+^ curve flipped polarity with respect to the CD′2–X^1^Σ^+^ input (associated with the upper diabat), while the D′^1^Σ^+^–X^1^Σ^+^ (Diabat 2) curve followed the same polarity as the CD′1–X^1^Σ^+^ input (associated with the lower diabat), as expected in the 2-level system. Similar behavior was noticed by Brady *et al.*^70^ Since we are dealing with a 3-level system (composed of D′^1^Σ^+^, C^1^Σ^+^ and ^1^Σ^+^(VI) states) in reality, we corrected the polarity of the diabatic C^1^Σ^+^–X^1^Σ^+^ curve by flipping its sign and allowing it to follow the polarity of the CD′2–X^1^Σ^+^ curve (associated with the upper diabat). Then the diabatic C^1^Σ^+^–X^1^Σ^+^ TDM used in the calculation is smoothed using cubic splines with *σ*_r_ = 0.096 a.u., after the diabatization procedure. Similarly to the previously discussed cases, the D′^1^Σ^+^–X^1^Σ^+^ curve (Diabat 2) obtained directly from the diabatization process exhibits a jump at the avoided crossing, possibly due to numerical errors arising from the simplification of the 3-level system to a 2-level system. Therefore, the curve was also smoothed out using cubic splines with *σ*_r_ = 0.067 a.u., denoted D′^1^Σ^+^–X^1^Σ^+^ (Diabat 2)-fitted. The relative intensity of the diabatic C^1^Σ^+^–X^1^Σ^+^ bands, which is the ratio of the first two bands intensities, is 0.034, which agrees well with the experimental values of Imhof *et al.*^94^ (RI = 0.029), and Chan *et al.*^95^ (RI = 0.031).

### 3.3 Rovibronic calculations

In this section, we evaluate the accuracy of the diabatic states and corresponding transitions obtained in the previous section, through spectral comparisons with the literature. At the same time, the CO total internal partition functions (TIPS) comparison with the values of Gamache *et al.*^102^ and Barklem and Collet^103^ with the absolute difference for each dataset [*Q*_Thiswork_–*Q*_Ref_], is shown in the ESI,† Section S1. Our data agree well with the TIPS values of Gamache *et al.*^102^ with a maximum relative error percentage of 1.328% at 9000 K, which corresponds to an absolute difference of −160.772. Barklem and Collet^103^ data also agreed well with our data with a maximum relative error percentage of 2.137% at 9000 K, corresponding to an absolute difference of −260.772.

#### 3.3.1 A^1^Π–X^1^Σ^+^ band system

**Fig. 9:**
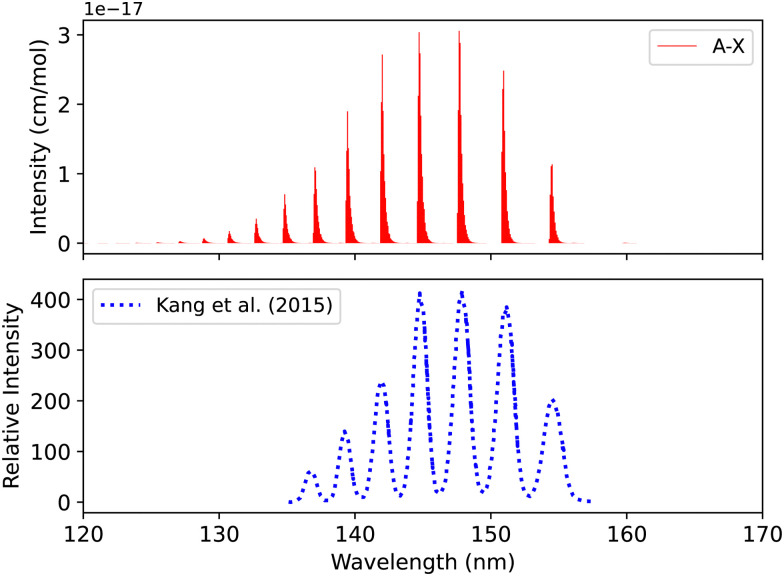
Spectrum of A^1^Π–X^1^Σ^+^ band: upper plot, our calculation; lower plot, experiment of Kang *et al.*^104^

The A^1^Π–X^1^Σ^+^ transition line list was computed by solving the Schrödinger equation on a grid of internuclear distances from 0.9 to 3.5 Å using Duo. Kang *et al.*^104^ conducted an experiment using the inelastic X-ray scattering (IXS) method with a resolution of 0.070 eV. The gas cell pressure was set to 9.75 × 10^−6^ bar with an uncertainty of 0.52%. The uncertainties in the experiment come mainly from the least-squares fitting and the calibration process. In our simulation, we assumed a temperature of 500 K, since the temperature was not given, and pressure of 9.75 × 10^−6^ bar to best compare with the experiment (Fig. 9). There is good agreement with the experiment regarding line position and intensity profile with an energy shift (0.01 < Δ*λ* < 0.044 nm) for the first six peaks with respect to the experiment.

**Fig. 10:**
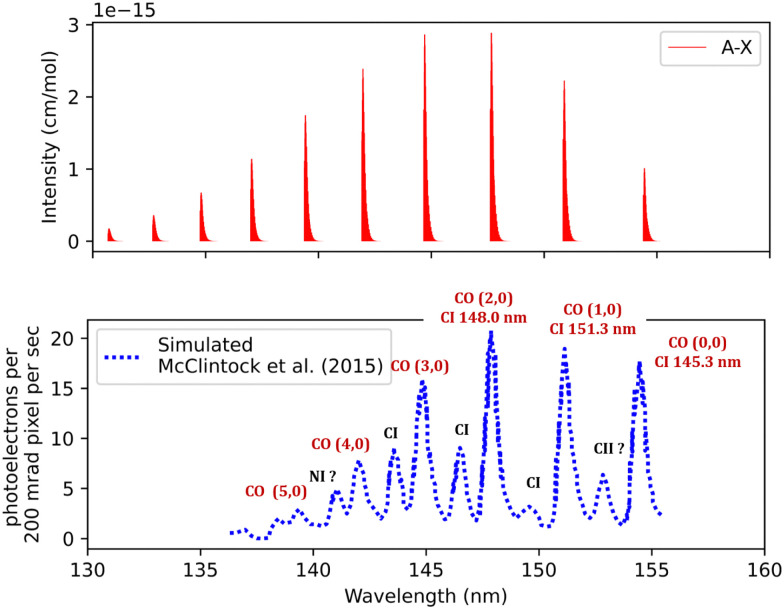
Fourth positive band system spectra of CO: upper plot, our simulation; lower plot, observed far ultraviolet spectrum of the Mars airglow observed by MAVEN during a periapsis limb observation with 110 km tangent altitude.^105^

The synthetic spectra obtained were also compared with the simulated normal-mode far ultraviolet spectra of the Mars airglow observed by MAVEN during a periapsis limb observation at 110 km tangent altitude. The normal mode was developed using imaging performance and radiometric response curves with atmospheric models to predict the instrument response,^105^ see Fig. 10. The first five bands coincide with the MAVEN IUVS peaks. The peak at 152.9 nm most probably corresponds to C II, while the one at 141.2 nm is likely associated with N I. This identification was made using NIST.^82^ Additionally, the 149.3, 146.3, and 145.2 nm peaks originate from C I emissions.^18^ The spectrum was calculated at 200 K and 0.008 bar (Fig. 10). There is good agreement with the observations regarding line position and intensity profile with a line position difference of (0.002 < Δ*λ* < 0.25 nm) for the first five peaks for the simulated spectra.

**Fig. 11:**
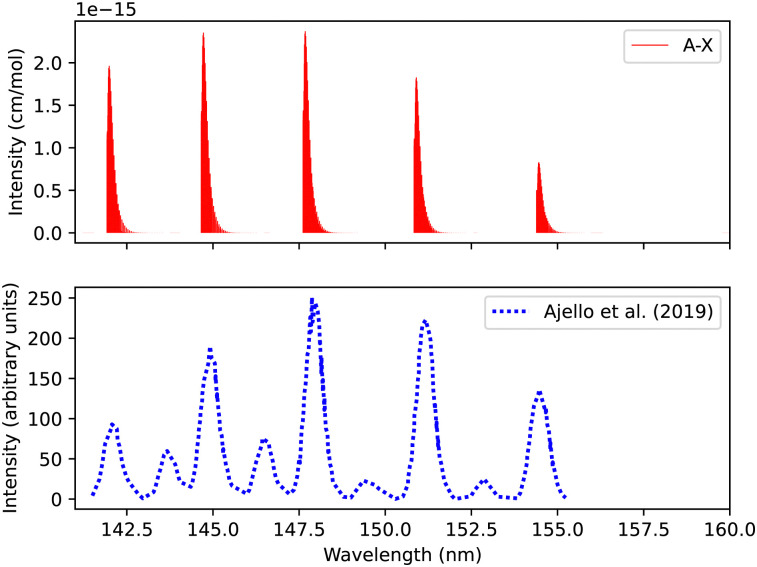
Spectrum of A^1^Π–X^1^Σ^+^ transition: upper plot, our calculations; lower plot, experiments due to Ajello *et al.*^18^

Fig. 11 shows a total experimental spectrum measured by Ajello *et al.*,^18^ using electron-impact-fluorescence. The gas pressure was set to 9.066 × 10^−9^ bar and at a temperature of 300 K. The comparison was made with the spectrum generated at 30 eV electron energy. The experimental setup consists of Mars Atmosphere and Volatile EvolutioN (MAVEN) mission imaging ultraviolet spectrograph optical engineering unit and an electron gun inside a large vacuum chamber. The line position difference between the experimental and our simulation is (0.0007 < Δ*λ* < 0.3 nm) for the first five peaks.

#### 3.3.2 The B^1^Σ^+^–X^1^Σ^+^ band system

**Fig. 12:**
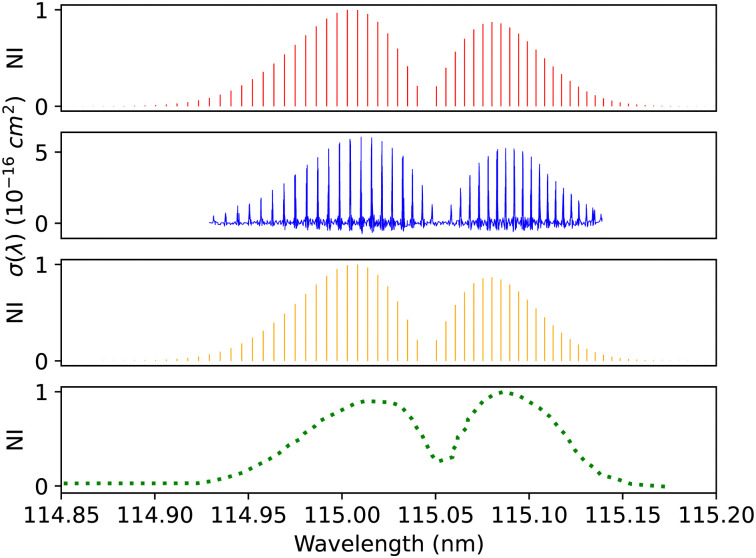
B^1^Σ^+^–X^1^Σ^+^ (0–0) spectrum: top panel, synthetic band calculated at Eidelsberg *et al.*^41^ experimental conditions. Second panel, calibration (0–0) band due to Eidelsberg *et al.*^41^ Third panel, synthetic band calculated at Federman *et al.*^106^ experimental conditions. Bottom panel, (0–0) band due to Federman *et al.*^106^ NI denotes normalized intensity.

The diabatic (0–0) band of the B^1^Σ^+^–X^1^Σ^+^ transition was compared with the (0–0) calibration band measured using UV spectroscopy at the SOLEIL synchrotron. The calibration band was fitted using the least-squares fitting method that fits the line position and the strength and width of the rotational transitions without assuming the molecular parameters. The CO gas was set to a temperature of 295 K and a pressure of 10^−4^ bar. The lines calibrated with uncertainty of 0.05 cm^−1^.^41^ Overall, there is excellent agreement in the line position (0.001 < Δ*λ* < 0.007 nm) and intensity profile, see Fig. 12.

**Fig. 13:**
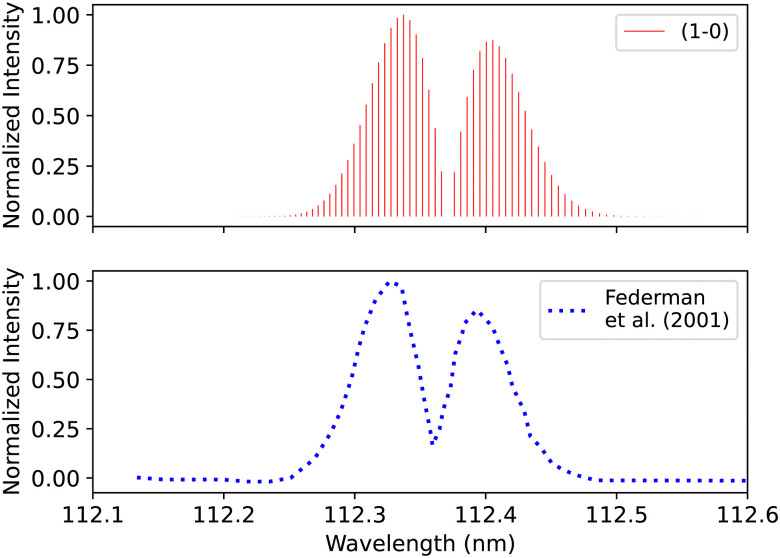
B^1^Σ^+^–X^1^Σ^+^ (1–0) spectrum: upper plot, synthetic band; lower plot, (1–0) band due to Federman *et al.*^106^

Federman *et al.*^106^ conducted an experiment using synchrotron radiation to measure the transmission spectra of the B^1^Σ^+^–X^1^Σ^+^ (0–0) and B^1^Σ^+^–X^1^Σ^+^ (1–0) bands. The average simulation temperature was set to 275 K because the temperature range in the experiment is between 250 K and 300 K. The pressure was set to 4 × 10^−6^ bar for the B^1^Σ^+^–X^1^Σ^+^ bands to match the experimental conditions. The uncertainty in pressure is (5%), and (1%) in the excitation temperature. The line position difference for the (0–0) band is (0.001 < Δ*λ* < 0.018 nm) (Fig. 12), and (0.01 < Δ*λ* < 0.017 nm) for (1–0) band (Fig. 13).

#### 3.3.3 The C^1^Σ^+^–X^1^Σ^+^ band system

**Fig. 14:**
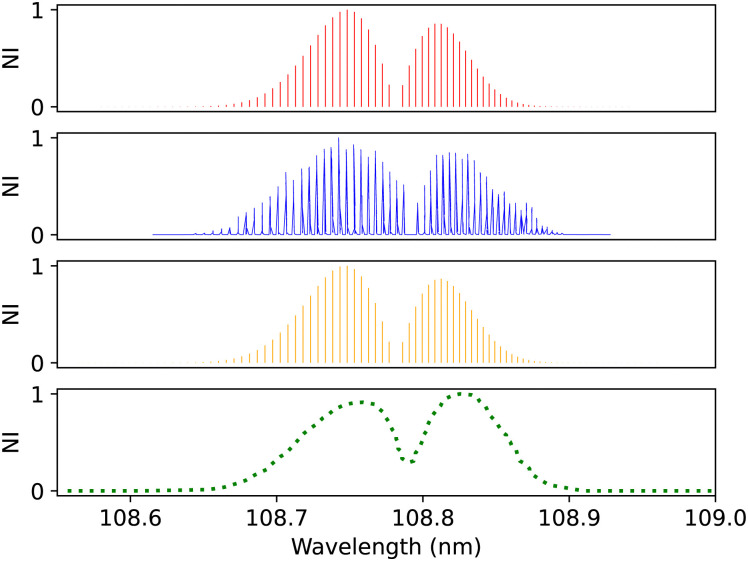
C^1^Σ^+^–X^1^Σ^+^ (0–0) spectrum: top panel, synthetic band calculated at Ubachs *et al.*^107^ experimental conditions. Second panel, calibration (0–0) band due to Ubachs *et al.*^107^ Third panel, synthetic band calculated at Federman *et al.*^106^ experimental conditions. Bottom panel, (0–0) band due to Federman *et al.*^106^ NI denotes normalized intensity.

Ubachs *et al.*^107^ measured an accurate spectrum of the C^1^Σ^+^–X^1^Σ^+^ (0,0) band using the VUV laser source in combination with 1 VUV/1 UV photoionization. The VUV spectrum linewidth is 0.4 cm^−1^, and the signal-to-noise accuracy is better than 0.150 cm^−1^. A possible source of error includes intensity noise caused by non-linearity in the wavelength scan and a Doppler shift due to misalignment of the crossed beams. The calibration accuracy of the frequency scale is 0.08 cm^−1^ in the vacuum ultraviolet. The instrument's high resolution allowed for observing R(32) and P(33) rotational lines at a low temperature of 250 K. Fig. 14 shows the diabatic C^1^Σ^+^–X1Σ^+^ (0,0) band calculated in this work in comparison with Ubachs *et al.*^107^'s measurements at 250 K and 3.99967 × 10^−3^ bar. Overall, there is good agreement in the line position (0.0003 < Δ*λ* < 0.006 nm) and intensity profile.

**Fig. 15:**
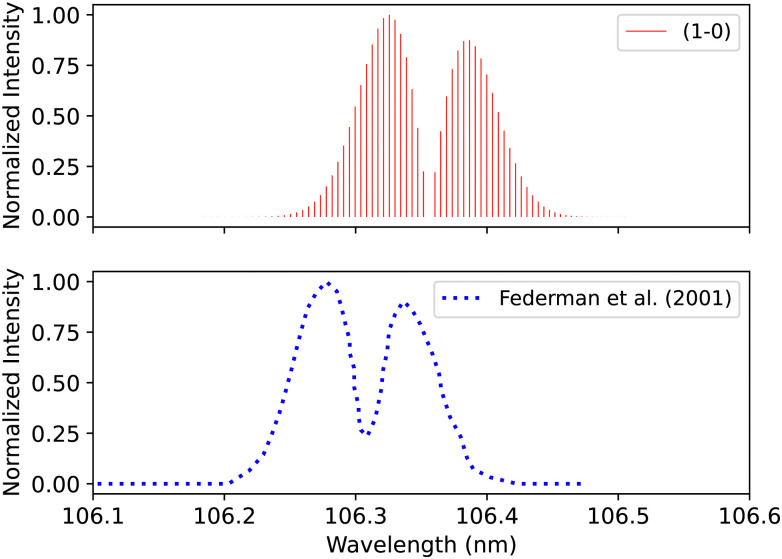
C^1^Σ^+^–X^1^Σ^+^ (1–0) synthetic band compared with the (1–0) band measured by Federman *et al.*^106^

Federman *et al.*^106^ also measured the transmission spectra of C^1^Σ^+^–X^1^Σ^+^ (0–0) and C^1^Σ^+^–X^1^Σ^+^ (1–0) bands at 275 K. The pressure for the (0–0) band (Fig. 14) is set to 8 × 10^−7^ bar and for the (1–0) band, to 4 × 10^−6^ bar. The line position difference of the (0–0) band is (0.01 < Δ*λ* < 0.013 nm). They found that the signal-to-noise ratio for the (1–0) band is lower than that of (0–0), and the quality of the fit is offset to 1.03 compared to the quality of the fit for (0–0), 1.02. According to Federman *et al.*^106^ the fitting quality reflects the difference between the observed spectrum and the least-squares fitting process conducted using the line wavelength positions identified in Tilford and Simmons^108^ study. The higher noise in this case is because the LiF cutoff is near the band position, and this might be one of the reasons for having a slight difference to our synthetic band, (Fig. 15), (Δ*λ* < 0.05 nm).

#### 3.3.4 The E^1^Π–X^1^Σ^+^ band system

The E^1^Π–X^1^Σ^+^ spectrum was computed in the adiabatic representation since only three states in *B*_2_ symmetry were obtained in the MCSCF/CASSCF calculations, so the upper ^1^Π state, which has an avoided crossing with the E^1^Π state, was not obtained.

**Fig. 16:**
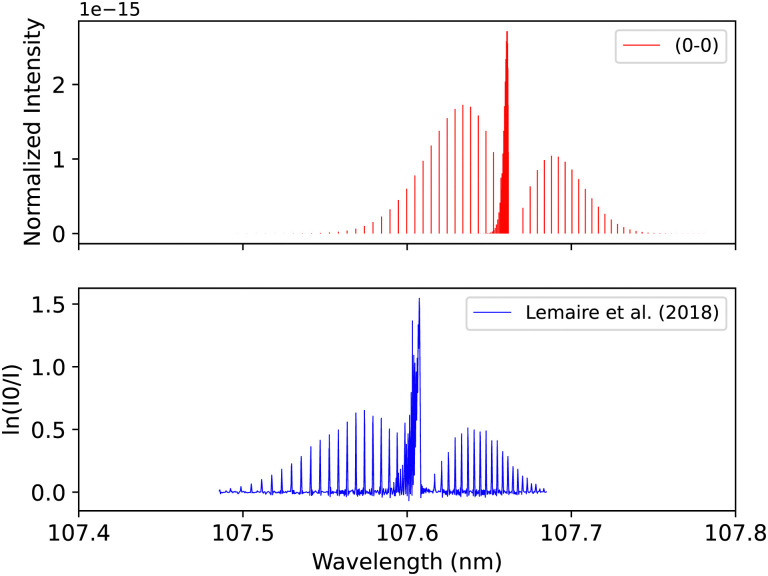
E^1^Π–X^1^Σ^+^ (0–0) synthetic band compared with the (0–0) band measured by Lemaire *et al.*^109^

Lemaire *et al.*^109^ recorded the spectra of E^1^Π–X^1^Σ^+^ transition using a Fourier-transform spectrometer connected to SOLEIL synchrotron, which provides resolving power up to 10^6^ in the energy range between 8 to 13 eV. The overall uncertainty in the line position is 0.01 cm^−1^. The spectrum for the (0–0) band was measured at 295 K after using Cacciani and Ubachs,^100^ Cacciani *et al.*,^110^ Daprà *et al.*^111^ bands as calibration peaks in order to reduce the possible source of error, which is associated with the calibration of the wavenumber scale by a small number of atomic transitions. The accuracy of the rotational line position depends on the absolute frequency calibration of the atomic lines and the spectral resolution and the strength of each line measured. Our adiabatic spectrum is calculated at 250 K and 1 bar pressure and is shifted by around 0.05 nm to the shorter wavelengths, see Fig. 16. This shift is probably due to the exclusion of the non-adiabatic couplings, and diagonal BO correction (DBOC) in the adiabatic representation, as discussed in the numerical equivalence of diabatic and adiabatic representations in the CH molecule by Brady *et al.*^69^

#### 3.3.5 The A^1^Π–X^1^Σ^+^, B^1^Σ^+^–X^1^Σ^+^, C^1^Σ^+^–X^1^Σ^+^ and E^1^Π–X^1^Σ^+^ bands spectral comparison

**Fig. 17:**
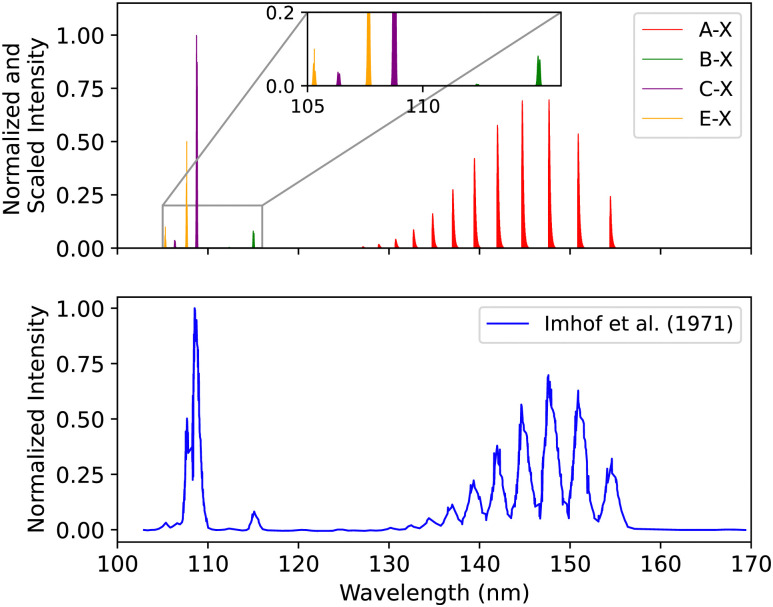
Spectral comparison of the A^1^Π–X^1^Σ^+^, B^1^Σ^+^–X^1^Σ^+^, C^1^Σ^+^–X^1^Σ^+^ and E^1^Π–X^1^Σ^+^ bands with the experiment of Imhof *et al.*^94^

A spectral comparison for the A^1^Π–X^1^Σ^+^, B^1^Σ^+^–X^1^Σ^+^, C^1^Σ^+^–X^1^Σ^+^ and E^1^Π–X^1^Σ^+^ bands was conducted using an energy loss spectroscopy measurement by Imhof *et al.*^94^ The experiment used an optical detector with a Kodak Wratten 2E filter. The upper vibrational levels were selected using a high-resolution inelastic electron energy analyzer that can discriminate against the other allowed transitions. The electron beam energy was set to 300 eV with an energy spread of 0.060 eV at a pressure of 6.666 × 10^−6^ bar. Since the intensity axis is given in arbitrary units, it was scaled based on the highest value of our theoretical intensity value. The line position difference for the first seven bands of the A^1^Π–X^1^Σ^+^ transition is 0.007 < Δ*λ* < 0.110 nm. For the first two peaks of the B^1^Σ^+^–X^1^Σ^+^ transition it is 0.041 < Δ*λ* < 0.066 nm, for C^1^Σ^+^–X^1^Σ^+^ it is Δ*λ* ≈ 0.2 nm, and for E^1^Π–X^1^Σ^+^ it is Δ*λ* ≈ 0.04 nm (Fig. 17).

Finally, Chan *et al.*^95^ used a high-resolution (0.048 eV FWHM) dipole (e, e) spectrometer to record the electron energy loss spectrum from 7 to 20.3 eV energy regime. The uncertainty in the experimental absolute optical oscillator strengths is 5% for the resolved peaks in the spectrum.

The energy shift for the first seven bands of the A^1^Π–X^1^Σ^+^ transition is 0.007 < Δ*λ* < 0.13 nm. For the first two peaks of the B^1^Σ^+^–X^1^Σ^+^ transition it is 0.015 < Δ*λ* < 0.054 nm, for C^1^Σ^+^–X^1^Σ^+^ it is 0.007 < Δ*λ* < 0.042 nm, and for E^1^Π–X^1^Σ^+^ 0.005 < Δ*λ* < 0.05 nm (see ESI,† Section S3 and Fig. S2).

### 3.4 The effect of diabatization on the synthetic spectra

#### 3.4.1 The B^1^Σ^+^–X^1^Σ^+^ band system

**Fig. 18:**
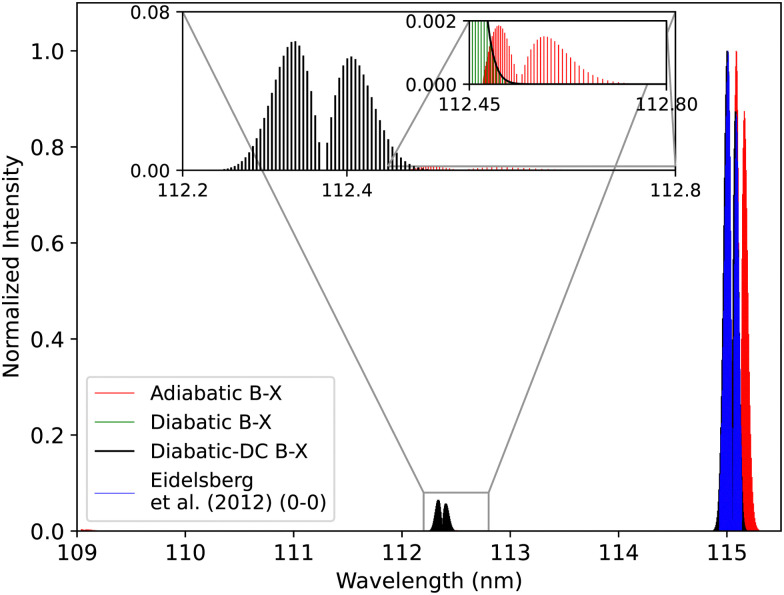
Comparison between the adiabatic and diabatic representations of the B^1^Σ^+^–X^1^Σ^+^ band system, and with Eidelsberg *et al.*^112^ experiment.

**Fig. 19:**
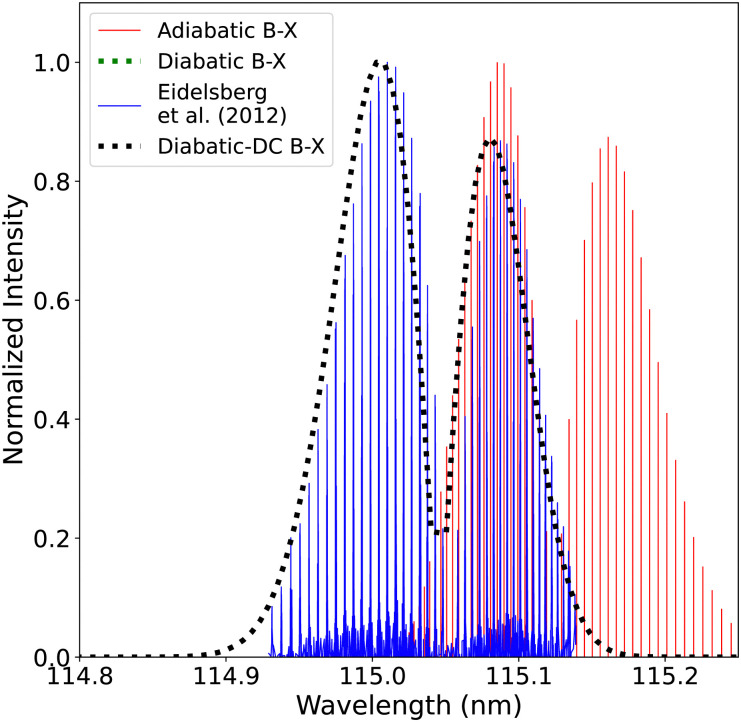
Comparison between the adiabatic and diabatic representations of the (0–0) band of B^1^Σ^+^–X^1^Σ^+^ band system and with Eidelsberg *et al.*^112^ experiment.

Theoretically, both adiabatic and diabatic representations should yield the same results when the non-adiabatic couplings (NACs) and diabatic couplings are fully taken into account.^69^ However, adiabatic models often omit the NACs to reduce the computational cost.^74^ The adiabatic spectrum of CO has been computed using adiabatic potential energy curves ranging from 0.9 Å to 1.26 Å, fitted and shifted to experimental parameters as mentioned in Section 2.3 and adiabatic transition dipole moment for B^1^Σ^+^–X^1^Σ^+^ and C^1^Σ^+^–X^1^Σ^+^ transitions. The effect of the diabatization is studied by comparing the adiabatic spectrum with the diabatic one (obtained using the processed curves as explained in Section 3.2, which extend from 0.9 Å to 1.44 Å) with/without DCs. Fig. 18, illustrates the diabatic effects on the computed spectra for the B^1^Σ^+^–X^1^Σ^+^ system while showing a comparison with a high-resolution spectrum of Eidelsberg *et al.*^112^ There are noticeable differences between the two computed spectra: the (0–0) peak shows a shift of about 0.08 nm in the line position, while the second peak position is shifted by about 0.174 nm, see Fig. 19.

The adiabatic peaks generally have lower intensity than the diabatic ones because of the repulsive character of the adiabatic potential energy curves after the adiabatic barrier. This repulsive character is due to tunneling into the continuum region after the adiabatic barrier. Certain energy levels are “stolen” or absent beyond the adiabatic barrier, and this causes intensity reduction; hence, the repulsive effect. This issue of intensity reduction can be solved by including NACs in the adiabatic representation or using the diabatic representation.^70^ The spectra obtained using the DCs in the diabatic representation almost overlap with that of the diabatic without the DCs since the value of the diabatic coupling is very small with a maximum value of 0.0124 cm^−1^. The regions of low intensity are sensitive and highly affected by the *ab initio* results and the profile of the couplings between the diabatic and the adiabatic representation.^74^

#### 3.4.2 The C^1^Σ^+^–X^1^Σ^+^ band system

**Fig. 20:**
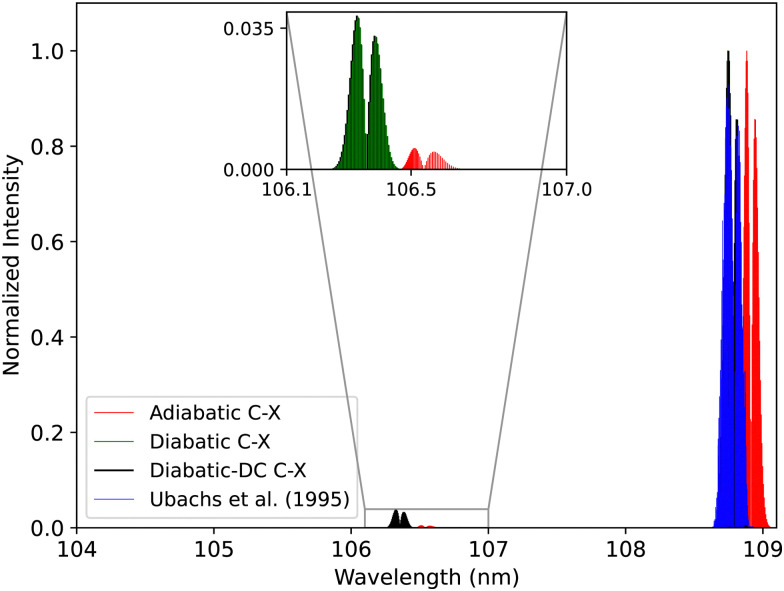
Comparison between the adiabatic and diabatic representations of the C^1^Σ^+^–X^1^Σ^+^ band system and with Ubachs *et al.*^107^ experiment.

**Fig. 21:**
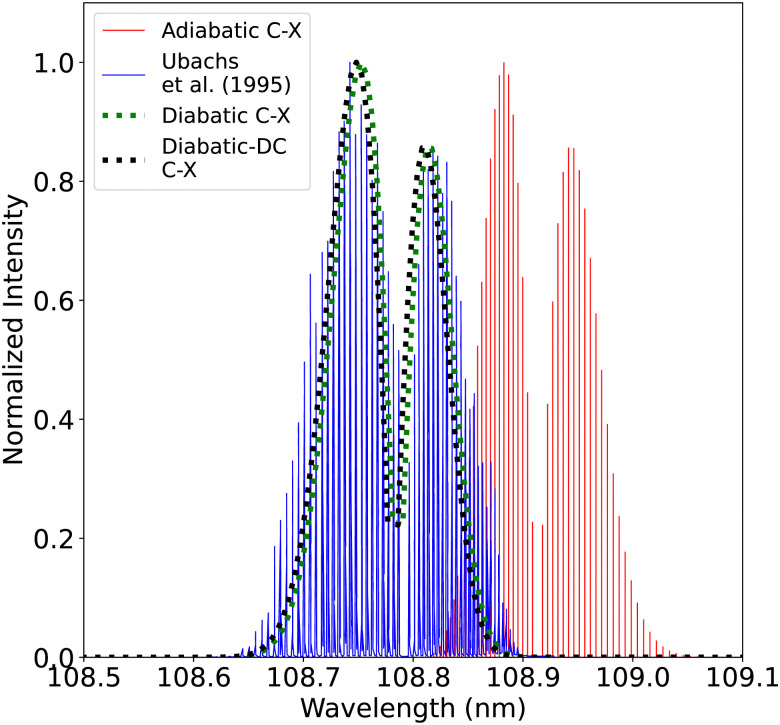
Comparison between the adiabatic and diabatic representations of the (0–0) C^1^Σ^+^–X^1^Σ^+^ band and with Ubachs *et al.*^107^ experiment.

Fig. 20, illustrates the computed spectra of the C^1^Σ^+^–X^1^Σ^+^ system in both representations while showing a comparison with the experimental spectra of Ubachs *et al.*^107^ Like for the B^1^Σ^+^ state, the PECs that were used in this comparison were fitted and shifted as described in Section 2.3 (adiabatic representation) and Section 3.2 (diabatic representation). The first peak is shifted by about 0.137 nm (Fig. 21), and the second peak by about 0.187 nm with respect to the band positions of Federman *et al.*^106^ This similarity is expected as the C^1^Σ^+^ state has deeper potential well before the adiabatic barrier. Therefore, the states involved with the measured transitions are further from the region of strong non-adiabatic couplings, unlike the case of the B^1^Σ^+^ state. The intensity of the (1–0) band is enhanced in the diabatic representations in a similar manner as in the BD′ system. The spectra obtained using the DCs in the diabatic representation gave better results than the diabatic one without the DCs. The line position difference is <0.0048 nm for the (0–0) peak and 0.0034 for the (1–0) peak. The difference here is more visible than in the BD′ system since the maximum value of diabatic couplings is much higher (975.6121 cm^−1^).

## 4 Conclusions

In summary, we present accurate potential energy curves for the ground and excited electronic states of CO, which were obtained by employing the CASSCF/MRCI+Q method with the aug-cc-pV5Z basis set. Our calculations yield spectroscopic constants exhibiting remarkable consistency with experimental values. The diabatic model is much better than an adiabatic model without non-adiabatic couplings; its use gives enhanced accuracy of line positions and intensities.

To validate our theoretical findings, we have compared the synthetic absorption spectra A^1^Π–X^1^Σ^+^, B^1^Σ^+^–X^1^Σ^+^, C^1^Σ^+^–X^1^Σ^+^, D′^1^Σ^+^–X^1^Σ^+^ and E^1^Π–X^1^Σ^+^ with the existing experimental spectra documented in the literature. We note that, unlike several of the experimental studies with which we compare, our calculations give absolute transition intensities. The high-resolution spectra presented in this paper can be used in forward and general circulation models to calculate the molecule's abundance in planetary atmospheres. Retrieving the molecular densities is essential in understanding the properties of the planetary atmosphere.

Our potential energy and transition dipole moment curves alongside a MARVEL (Measured Active Rotation Vibration Energy Levels)^113^ study of the rovibronic transitions of CO, which is currently nearing completion,^114,115^ will be used to generate an ExoMol-style^81^ hot line list for CO. These curves can also be used as the basis of a study of temperature-dependent photodissociation rates^116^ and predissociation effects.^117,118^

## Data availability

Sample data/input files from this work are available in the ESI,† named Supplementary Information.pdf and Supplementary Information.zip. The ESI† complements the main paper by providing additional details and data pertinent to the study. Supplementary Information.pdf encompasses the following datasets: partition function (S1): this section presents a comparison of CO partition functions with values sourced from references, providing a comprehensive overview. Spectral comparisons (S2): a comparative analysis of spectra for A–X, B–X, C–X, and E–X transitions, compared with experimental data sourced from Chan *et al.* Supplementary Information.zip includes: (1) sample Duo input files corresponding to all the transitions mentioned in the text, at different temperature and pressure conditions (.inp format). (2) A sample ExoCross input file of the A–X transition (.inp format). (3) Grids of the PECs and TDMCs as extracted from the MOLPRO output (.csv format). (4) Grids of the original (*ab initio*) and fitted data (as introduced in Duo) for the A, B, and C states (.csv format). (5) Grids of the stick spectra for all the transitions mentioned in (1) (.xlsx format). (6) Suggested fitting constants for the A, B, and C adiabatic states used in the manuscript (.pdf format). (7) A “read-me” file with more details about all available data. Codes Duo and ExoCross are freely available on the ExoMol github page https://github.com/ExoMol.

## Conflicts of interest

There are no conflicts to declare.

## Supplementary Material

CP-027-D4CP03418J-s001

CP-027-D4CP03418J-s002
