# Supplementary material for: Theoretical investigation of the A1Π–X1Σ+, B1Σ+–X1Σ+, C1Σ+–X1Σ+, and E1Π–X1Σ+ transitions of the CO molecule
Source: Phys Chem Chem Phys. 2024 Dec 27;27(5):2783–801. doi: 10.1039/d4cp03418j (PMC11736852; doi:10.1039/d4cp03418j)
Supplement: CP-027-D4CP03418J-s001 [file CP-027-D4CP03418J-s001.pdf]

Supplementary Information (SI) for Physical Chemistry Chemical Physics.
This journal is © the Owner Societies 2024

Theoretical investigation of the $A^1\Pi$ – $X^1\Sigma^+$, $B^1\Sigma^+$ – $X^1\Sigma^+$, $C^1\Sigma^+$ – $X^1\Sigma^+$ and $E^1\Pi$ – $X^1\Sigma^+$ transitions of the CO molecule

Supplementary materials

Malathe Khalil,[a] Salman Mahmoud,[b] Ryan P. Brady,[c] Mubarak Almehairbi, [d] Marko Gacesa,[b] Sergei N. Yurchenko,[c] Jonathan Tennyson,[c] Amal Al Ghaferi,[*a] and Nayla El-Kork[*b,e]

[a] Department of Mechanical Engineering, Khalifa University, Abu-Dhabi, United Arab Emirates.
[b] Physics Department, Khalifa University, Abu-Dhabi, United Arab Emirates.
[c] Department of Physics and Astronomy, University College London, London, WC1E 6BT, United Kingdom.
[d] Chemistry Department, Khalifa University, Abu-Dhabi, United Arab Emirates.
[e] Planetary Science Center, Khalifa University, Abu-Dhabi, United Arab Emirates; E-mail: nayla.elkork@ku.ac.ae

S1. Partition function

The partition functions of the CO are computed with ExoCross program using the summation over energy levels ($\tilde{E}_n$) from our calculated line list using the following equation:

$$Q(T) = \sum_n g_n^{tot} e^{-\frac{c_2 \tilde{E}_n}{T}}$$

Here, T is the temperature in (K), g_n^{tot} is the total degeneracy that is a function of the nuclear-spin statistical weight factor (g_n^{ns}) and rotational quantum number (J_n), ($g_n^{tot} = g_n^{ns}(2J_n + 1)$ [1].

The computed partition function is then compared to Gamache et al. [2] and Barklem and Collet [3] datasets up to 10000 K temperature range (Figure 1). Our data agree well with the total Internal Partition Sums partition functions by Gamache et al. [2], which covers temperatures up to 9000 K with a maximum relative error percentage [(($Q_{our-data} - Q_{literature}$)/$Q_{literature}$)) ∗ 100] of 1.328 % at 9000 K. Barklem and Collet [3] data also agreed well with Gamache et al. [2] data up to 6000K, and with our data with a maximum relative error percentage of 2.137 % at 9000 K.

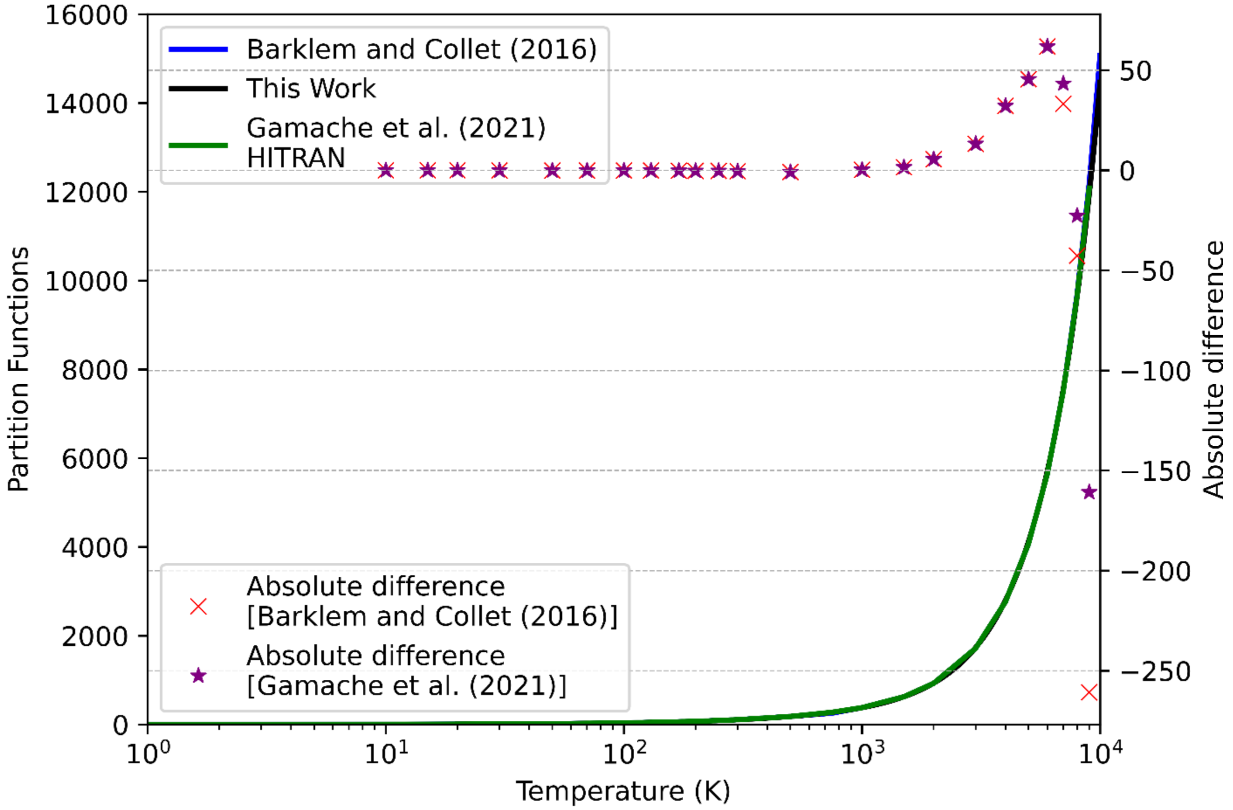

Figure 1S. CO partition functions comparison with the values of Gamache et al. [2] and Barklem and Collet [3] with the relative error percentage for each dataset.

S2. Different electronic configuration models

Table 1S. Different electronic configuration models

Basis set	Occupied Orbitals in CASSCF	Closed Orbitals in CASSCF	Number of close orbitals in CI	Number of core orbitals in CI	Number of States [A_1, B_1, B_2, A_2]
For O, C: aug-cc-pVQZ	8,2,2,0	0,0,0,0	0,0,0,0	0,0,0,0	3,2,2,2
For O, C: aug-cc-pVQZ	8,2,2,0	2,0,0,0	2,0,0,0	0,0,0,0	3,2,2,2
For O, C: aug-cc-pVQZ	8,2,2,0	3,0,0,0	3,0,0,0	0,0,0,0	3,2,2,2
For O, C: aug-cc-pVQZ	8,2,2,0	4,0,0,0	4,0,0,0	0,0,0,0	3,2,2,2
For O, C: aug-cc-pVQZ	8,2,2,0	4,0,0,0	4,0,0,0	4,0,0,0	3,2,2,2
For O, C: cc-pV5Z	8,2,2,0	0,0,0,0	0,0,0,0	0,0,0,0	3,2,2,2
For O, C: cc-pVTZ	8,2,2,0	0,0,0,0	0,0,0,0	0,0,0,0	3,2,2,2
For O, C: cc-pV5Z	8,2,2,0	0,0,0,0	0,0,0,0	0,0,0,0	3,2,2,2
For O, C: cc-pVQZ	8,2,2,0	0,0,0,0	0,0,0,0	0,0,0,0	3,2,2,2
For O, C: aug-cc-pV5Z	8,2,2,0	0,0,0,0	0,0,0,0	0,0,0,0	3,2,2,2
For O, C: cc-pV5Z	8,2,2,0	2,0,0,0	2,0,0,0	2,0,0,0	5,3,3,3
For O, C: cc-pV5Z	8,2,2,0	3,0,0,0	3,0,0,0	3,0,0,0	5,3,3,3
For O, C: cc-pV5Z	8,3,3,0	3,0,0,0	3,0,0,0	3,0,0,0	5,3,3,3
For O, C: cc-pV5Z	8,3,3,0	2,0,0,0	2,0,0,0	2,0,0,0	5,3,3,3
For O, C: cc-pV5Z	8,2,2,0	0,0,0,0	0,0,0,0	0,0,0,0	5,3,3,3
For O, C: cc-pV5Z	8,3,3,0	0,0,0,0	0,0,0,0	0,0,0,0	5,3,3,3
For O, C: aug-cc-pV5Z	8,3,3,0	2,0,0,0	2,0,0,0	2,0,0,0	6,3,3,2

For O, C: aug-cc-pV5Z	10,4,4,0	3,0,0,0	3,0,0,0	3,0,0,0	5,3,3,2
For O, C: *cc-pV6Z*	8,3,3,0	2,0,0,0	2,0,0,0	2,0,0,0	10,3,3,2
For O, C: aug-cc-pV5Z	8,3,3,0	2,0,0,0	2,0,0,0	2,0,0,0	9,3,3,3
For O, C: aug-cc-pVTZ	8,3,3,0	2,0,0,0	2,0,0,0	2,0,0,0	12,3,3,2
For O, C: cc-pV5Z	8,3,3,0	3,0,0,0	3,0,0,0	3,0,0,0	10,3,3,6
For O, C: cc-pV6Z	8,3,3,0	2,0,0,0	2,0,0,0	2,0,0,0	10,3,3,2
For O, C: aug-cc-pV6Z	8,3,3,0	2,0,0,0	2,0,0,0	2,0,0,0	8,3,3,2
For O: cc-pV5Z<br>For C: aug-cc-pVTZ	8,3,3,0	2,0,0,0	2,0,0,0	2,0,0,0	8,3,3,2
For O: aug-cc-pVTZ<br>For C: cc-pV5Z	8,3,3,0	2,0,0,0	2,0,0,0	2,0,0,0	8,3,3,2
For O: cc-pV5Z<br>For C: aug-cc-pVTZ	8,3,3,0	0,0,0,0	0,0,0,0	0,0,0,0	8,3,3,2
For O: aug-cc-pVTZ<br>For C: cc-pV5Z	8,3,3,0	0,0,0,0	0,0,0,0	0,0,0,0	8,3,3,2
For O: cc-pV5Z<br>For C: aug-cc-pVTZ	8,3,3,0	3,0,0,0	3,0,0,0	3,0,0,0	8,3,3,2
For O: aug-cc-pVTZ<br>For C: cc-pV5Z	8,3,3,0	3,0,0,0	3,0,0,0	3,0,0,0	8,3,3,2

S3. The spectral comparison for A-X, B-X, C-X, and E-X transitions with Chan et al., experiment.

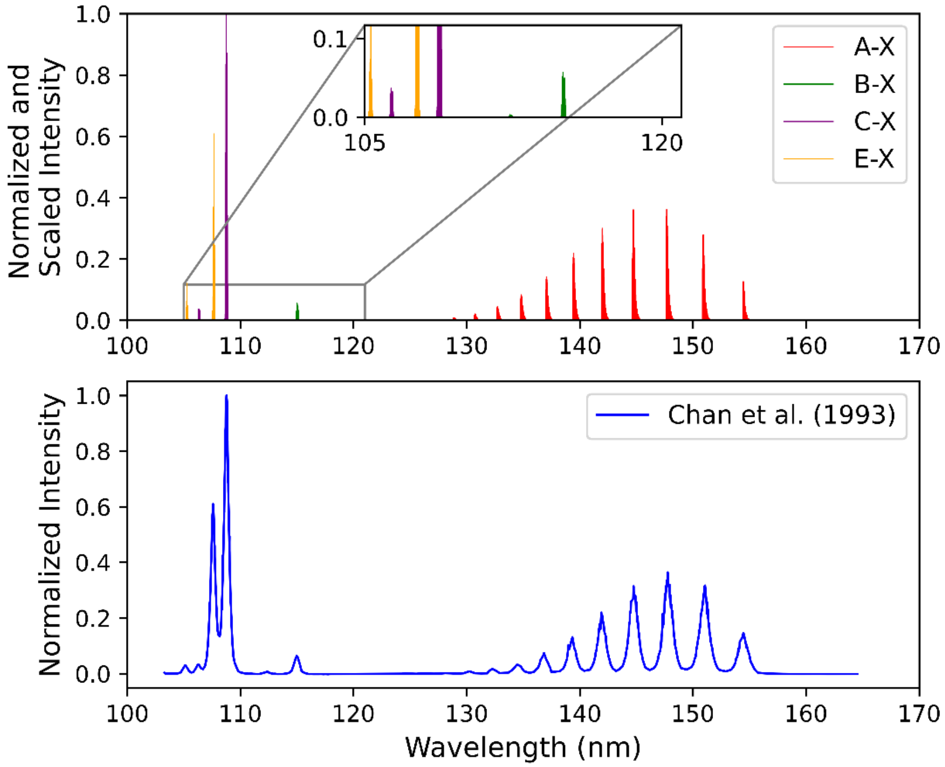

Figure 2S The spectral comparison for A-X, B-X, C-X, and E-X transitions with Chan et al., 1993 experiment [4].

S4. NACs and DCs before and after fitting.

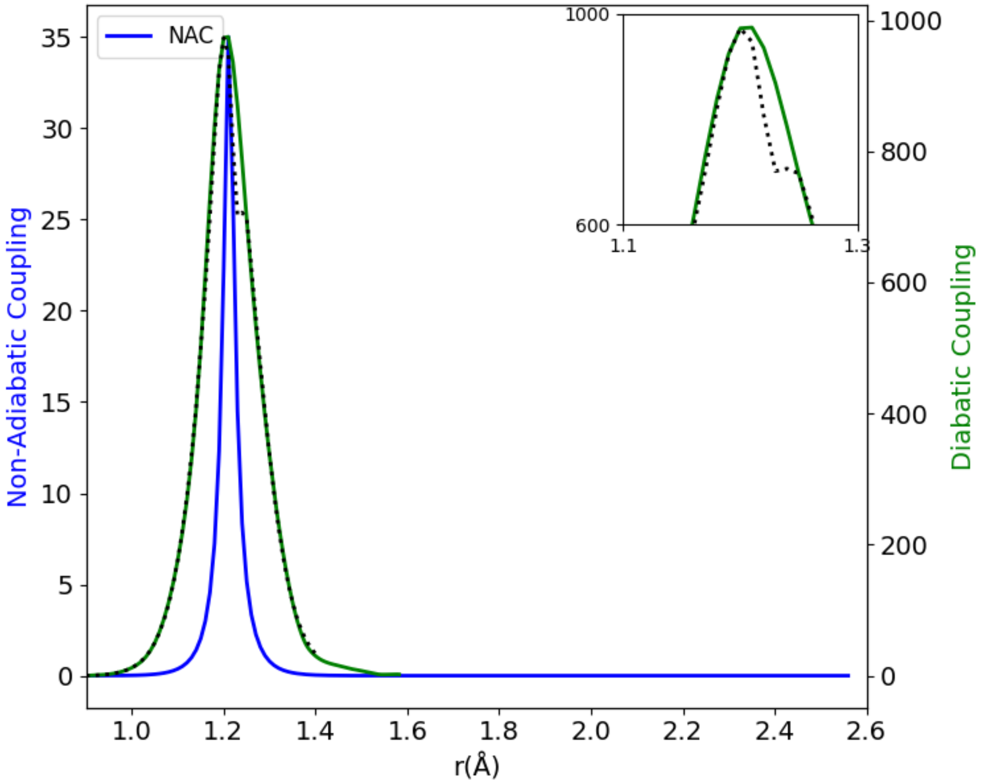

Figure 3S The NACs and DCs of the CD' system.

References:

(1) Yurchenko, S. N.; Al-Refaie, A. F.; Tennyson, J. EXOCROSS: A General Program for Generating Spectra from Molecular Line Lists. *Astron. Astrophys.* **2018**, *614*, 1–12. https://doi.org/10.1051/0004-6361/201732531.

(2) Gamache, R. R.; Vispoel, B.; Rey, M.; Nikitin, A.; Tyuterev, V.; Egorov, O.; Gordon, I. E.; Boudon, V. Total Internal Partition Sums for the HITRAN2020 Database. *J. Quant. Spectrosc. Radiat. Transf.* **2021**, *271*, 107713. https://doi.org/10.1016/j.jqsrt.2021.107713.

(3) Barklem, P. S.; Collet, R. Astrophysics Partition Functions and Equilibrium Constants for Diatomic Molecules and Atoms of Astrophysical Interest. *Astron. Astrophys.* **2016**, *96*, 588.

(4) Chan, W. F.; Cooper, G.; Brion, C. E. Absolute Optical Oscillator Strengths for Discrete and Continuum Photoabsorption of Carbon Monoxide (7-200 EV) and Transition Moments for the X 1Σ+ → A 1Π System. *Chem. Phys.* **1993**, *170* (1), 123–138. https://doi.org/10.1016/0301-0104(93)80098-T.
