# Supplementary material for: Theoretical investigation of the A1Π–X1Σ+, B1Σ+–X1Σ+, C1Σ+–X1Σ+, and E1Π–X1Σ+ transitions of the CO molecule
Source: Phys Chem Chem Phys. 2024 Dec 27;27(5):2783–801. doi: 10.1039/d4cp03418j (PMC11736852; doi:10.1039/d4cp03418j)
Supplement: CP-027-D4CP03418J-s002 [file CP-027-D4CP03418J-s002.zip › p.e.c_fitting_information.pdf]

## Potential Energy Curves fitting information

## $A^1\Pi$ state

## Effective Morse Oscillator parameters

| **Coefficient** | **Value** |
|---|---|
| Te | 65070.0 |
| Ae | 87750.0 |
| re | 1.245 |
| a0 | 3.437826775 |
| a1 | -1.4390756979 |
| a2 | -18.079875456 |
| a3 | 147.989719729 |
| a4 | 281.162932559 |
| a5 | -3079.766474149 |
| a6 | 1480.412775250 |
| a7 | 23516.882196800 |
| a8 | -35414.969313453 |
| a9 | -52709.832600099 |
| a10 | 123675.624624069 |
| a11 | 15990.0029343340 |
| a12 | -152667..669384484 |
| a13 | 79371.67 |

## $B^1\Sigma$ state

## Ninth-order polynomial Parameters

| Coefficient | Value |
|---|---|
| a0 | 4304348 |
| a1 | -8314711 |
| a2 | 4057177 |
| a3 | -11335826 |
| a4 | 30634333 |
| a5 | -19765572 |
| a6 | -14433848 |
| a7 | 24485256 |
| a8 | -11299152 |
| a9 | 1766956 |

## $C^1\Sigma$ state

## Ninth-order polynomial Parameters

| Coefficient | Value |
|---|---|
| a0 | -91253.72 |
| a1 | 6406024 |
| a2 | -11587182 |
| a3 | 451236.9 |
| a4 | 3891456 |
| a5 | 8674098 |
| a6 | -3333987 |
| a7 | -14040139 |
| a8 | 13032092 |
| a9 | -3298812 |
